# Transcriptomic and Metabolomic Analysis Revealed Roles of Yck2 in Carbon Metabolism and Morphogenesis of *Candida albicans*

**DOI:** 10.3389/fcimb.2021.636834

**Published:** 2021-03-16

**Authors:** Karl Liboro, Seong-Ryong Yu, Juhyeon Lim, Yee-Seul So, Yong-Sun Bahn, Hyungjin Eoh, Hyunsook Park

**Affiliations:** ^1^ Department of Biological Sciences, California State University, Los Angeles, CA, United States; ^2^ Department of Biotechnology, College of Life Science and Biotechnology, Yonsei University, Seoul, South Korea; ^3^ Department of Molecular Microbiology and Immunology, Keck School of Medicine, University of Southern California, Los Angeles, CA, United States

**Keywords:** yeast casein kinase 2, morphogenesis, hyphae formation, carbon metabolism, starvation

## Abstract

*Candida albicans* is a part of the normal microbiome of human mucosa and is able to thrive in a wide range of host environments. As an opportunistic pathogen, the virulence of *C. albicans* is tied to its ability to switch between yeast and hyphal morphologies in response to various environmental cues, one of which includes nutrient availability. Thus, metabolic flexibility plays an important role in the virulence of the pathogen. Our previous study has shown that *C. albicans* Yeast Casein Kinase 2 (CaYck2) regulates the yeast-to-hyphal switch, but its regulatory mechanisms remain unknown. This study further elucidated the role of Yck2 in governing morphology and carbon metabolism by analyzing the transcriptome and metabolome of the *C. albicans YCK2* deletion mutant strain (*yck2*Δ strain) in comparison to the wild type strain. Our study revealed that loss of CaYck2 perturbs carbon metabolism, leading to a transcriptional response that resembles a transcriptional response to glucose starvation with coinciding intracellular accumulation of glucose and depletion of TCA cycle metabolites. This shift in the metabolome is likely mediated by derepression of glucose-repressed genes in the Mig1/2-mediated glucose sensing pathway and by downregulation of glycolytic genes, possibly through the Rgt1-mediated SRR pathway. In addition, genes involved in beta-oxidation, glyoxylate cycle, oxidative stress response, and arginine biosynthesis were upregulated in the *yck2*Δ strain, which is highly reminiscent of *C. albicans* engulfment by macrophages. This coincides with an increase in arginine degradation intermediates in the *yck2*Δ strain, suggesting arginine catabolism as a potential mechanism of CaYck2-mediated filamentation as seen during *C. albicans* escape from macrophages. Transcriptome analysis also shows differential expression of hyphal transcriptional regulators Nrg1 and Ume6. This suggests dysregulation of hyphal initiation and elongation in the *yck2*Δ strain which may lead to the constitutive pseudohyphal phenotype of this strain. Metabolome analysis also detected a high abundance of methyl citrate cycle intermediates in the *yck2*Δ strain, suggesting the importance of CaYck2 in this pathway. Taken together, we discovered that CaYck2 is an integral piece of carbon metabolism and morphogenesis of *C. albicans*.

## Introduction

*Candida albicans* is a part of the commensal microbiota and can be found on skin as well as mucosal surfaces such as in the oral cavity, gastrointestinal tract, and urogenital area. As an opportunistic pathogen, *C. albicans* often causes fatal infections in immunocompromised populations, which is a growing threat to human health. The virulence of *C. albicans* is largely related to its pleomorphic nature—the ability to switch between yeast, pseudohyphae, and true hyphae forms. The morphology switch is triggered by a wide range of environmental cues including pH, temperature, and nutrient availability. Because *C. albicans* often faces different nutrient availability, it must be metabolically adaptable to survive in various host niches. In particular, flexible carbon metabolism plays a critical role in fitness and pathogenicity. For example, environmental glucose concentrations affect adhesion, stress resistance, and invasion of *C. albicans* in animal models, indicating that regulatory mechanisms of virulence are controlled by dynamic changes in glucose availability (Vargas et al., 1993; Rodaki et al., 2009; Ene et al., 2012; Ene et al., 2013). Similarly, *C. albicans *strains with defects in the glyoxylate cycle are avirulent in animal models (Lorenz and Fink, 2001).

Carbon metabolism is also intrinsically tied to hyphae formation. Hyphae formation is triggered by various nutritional cues including carbon sources, presence of serum or *N*-acetylglucosamine, and nitrogen sources (Landau et al., 1965; Holmes and Shepherd, 1987). In fact, glucose has been shown to be the active hyphae-inducing component in serum (Hudson et al., 2004). Similarly, expression of secreted aspartyl proteinases (SAPs) and hyphae formation are greatly increased in lower glucose concentrations through the adenylate cyclase pathway-mediated activation of Efg1 (Buu and Chen, 2014). In addition, several studies have highlighted the importance of glucose-sensing pathways in regulating morphogenesis in *C. albicans*. Glucose-sensing is mediated by three different overlapping signaling pathways: Glucose Repression (GR) pathway, Adenylate Cyclase (AC) pathway, and Sugar Receptor Repressor (SRR) pathway (Sabina and Brown, 2009). The GR pathway involves Mig1/2p repressing hexose transporter genes (HGTs) in the presence of high glucose. It has been shown that *mig1*/*2* mutants have defects in hyphal length (Lagree et al., 2020). The AC pathway involves adenylate cyclase (Cyr1) producing cyclic AMP (cAMP), which subsequently activates protein kinase A (PKA). Cyr1 can be activated by several upstream components such as Ras1 or the sugar-sensing G-protein coupled receptor (Gpr1). PKA activates the transcriptional regulator Efg1p, which regulates white-opaque switching and hyphae formation (Grahl et al., 2015; Silao et al., 2019). Lastly, SRR pathway involves the transcriptional repressor Rgt1, which regulates HGTs as well as glycolytic genes such as *TYE7* and *HXK2*. Rgt1 itself is regulated by the high affinity glucose sensor Hgt4p. Overexpression of *HGT4* or deletion of *RGT1* leads to hyper filamentation, which is believed to be caused by cross-talk between the SRR and the AC pathway (Sexton et al., 2007).

Our recent study found that a loss of *C. albicans* Yeast Casein Kinase 2 (CaYck2) function results in constitutive pseudohyphae formation and upregulation of hyphal-specific genes (HSGs), suggesting that CaYck2 serves as a negative regulator of hyphae formation. *YCK2* deletion mutant strain (*yck2*Δ* *strain) has significantly increased expression of *UME6, *a transcription factor involved in hyphal transition, and its downstream HSGs such as *ALS3* and *HWP1* (Jung et al., 2017). The *yck2*Δ* *strain also shows hypersensitivity to cell wall damaging agents and compensatory chitin deposit on the cell wall (Jung et al., 2017). These results suggest that CaYck2p plays an important role in maintaining cell wall integrity and the morphogenic switch from yeast to hyphae. However, it remains elusive how CaYck2 is functionally connected to regulatory pathways governing morphogenesis and how it affects the transcriptional landscape of HSGs in *C. albicans*.

CaYck2 is an orthologue of eukaryotic casein kinase 1 (CK1) family with significant amino acid similarity to two *Saccharomyces cerevisiae* yeast casein kinases, ScYck1 and ScYck2, which share redundant functions in regulating cell integrity and the budding process (Robinson et al., 1993). ScYck1 and ScYck2 function in the SRR pathway. Binding of glucose to the Rgt2/Snf3 glucose sensor triggers ScYck1/2-mediated phosphorylation of Std1/Mth1. Phosphorylation of the corepressors Std1/Mth1 targets them for degradation. The depletion of Std1/Mth1 prevents Rgt1 from binding to the promoters of hexose transporter genes, thus allowing expression of HGTs in the presence of glucose (Snowdon and Johnston, 2016). It is worth noting that deletion of Rgt2/Snf3 or Std1/Mth1 reduces mitochondrial efficiency in *S. cerevisiae* (Choi et al., 2015). In *C. albicans*, Hgt4 is the ortholog of the *S. cerevisiae* Rgt2/Snf3 glucose sensor while CaRgt1 acts similarly as the transcriptional repressor (Sexton et al., 2007). It is thus expected that CaYck2 is also involved in glucose sensing and carbon metabolism, but no formal studies have yet been performed to verify the function of CaYck2 in the SRR pathway.

This study investigated the regulatory mechanisms of CaYck2 in governing morphogenesis and cellular metabolism by analyzing the transcriptome and metabolome of the *yck2*Δ strain in comparison to the wild type strain. Because constitutive pseudohyphae formation and upregulation of HSGs in the *yck2*Δ* *strain is reminiscent of the hyphal growth stage of the wild type strain, we hypothesize that the transcriptome profile of the *yck2*Δ* *strain is likely similar to that of the wild type hyphal cells. Furthermore, because CaYck2 is homologous to ScYck1/2 in the SRR pathway, we hypothesize that CaYck2 has similar functions in glucose sensing, morphogenesis, and maintaining carbon metabolism. If glucose sensing is impacted, we suspect that the *yck2*Δ* *strain will also have significant changes in its metabolome. To test these hypotheses, we performed RNA-sequencing (RNA-seq) analysis to assess the expression of HSGs and metabolic genes as well as gas chromatography with mass spectroscopy (GC-MS) to assess any changes in the metabolome.

## Materials and Methods

### Strains and Media

*C. albicans *strains used in this study are listed in **Table 1**. All strains were stored in YPD (1% yeast extract, 2% peptone, 2% glucose [Fisher Bioreagents]) medium with 25% glycerol at −80°C. To recover the cells, the frozen stocks were streaked on YPD agar and incubated at 30°C for 2 days. A single colony was then inoculated in YPD broth and grown at 30°C for overnight to obtain fresh yeast cells. To induce hyphae formation, fresh cultures were washed with phosphate buffered saline (PBS, 0.8% NaCl, 0.02% KCl, 0.144% Na_2_HPO_4_, 0.024% KH_2_PO_4_, adjusted to pH 7.2) and 1 × 10^6^ cells/mL yeast cells were transferred to RPMI 1640 medium (Hyclone) and incubated for 3 h at 37°C. All work with *C. albicans* followed institutional guidelines for work with Biohazardous material.

**Table 1:** Strains used in this study.

Strains	Genotype	Reference
DIC 185	*ura3Δ::λimm434::URA3-IRO1/ura3Δ::λimm434 arg4::hisG::ARG4/arg4::hisG his1::hisG::HIS1/his1::hisG*	Phan et al. (2013)
JAC1401U	*yck2Δ::HIS1/yck2Δ::ARG4, his1/his1, arg4/arg4, URA3/ura3*	Jung et al. (2017)
JAC14010	*yck2Δ::HIS1/yck2Δ::ARG4, his1/his1, arg4/arg4, ura3::URA3-YCK2/ura3*	Jung et al. (2017)

### RNA Sequencing and Transcriptome Analysis

*C. albicans* strains grown overnight in YPD medium were transferred to 50 mL of fresh YPD medium and incubated at 30°C until the OD_600_ reached 0.6. The cells were then harvested by centrifugation and lyophilized. Total RNAs were isolated by Easy-BLUE (iNtRON), treated with DNase I (Qiagen), and purified with RNeasy MiniElute clean up kit (Qiagen) following the manufacturer’s instruction. Three biologically independent cultured samples were prepared for each strain. The cDNA library was constructed with the 1 µg of total RNAs for each sample by Illumina TruSeq RNA library kit v2 (Illumina) and sequenced by Illumina platform. The adapter sequences were trimmed from the sequencing reads and slow-complexity or low-quality sequence were masked by using Cutadpat v2.4 with Python 3.5.2. (Martin, 2011). The reference genome sequence of *Candida albicans* SC5314 and annotation data were downloaded from the NCBI ftp server, and the reads were aligned to the genome sequence using Hisat2 v2.1.0 with the Hisat and Bowtie2 algorithm and processed as previously reported  (Pertea et al., 2016). Hisat2 was performed with “-p 30” and “– dta -1” option and other parameters set as default. Aligned reads were converted and sorted using Samtools v0.1.19  (Li et al., 2009) with “-Sb -@ 8” option for converting, and “-@ 20 –m 2000000000” option for sorting and the other parameters set as default. Transcript assembly and abundance estimation were performed by Stringtie v1.3.6 by using “-p 12” option, and also “-B” option to run the Ballgown analysis  (Frazee et al., 2015). Assembled transcripts were merged to single GTF file, and the relative transcript abundances were calculated *via* Fragments Per Kilobase of exon per Million fragments mapped (FPKM). The FPKM and read count matrix was generated by python script “prepDE.py” and analyzed by DESeq2. Differentially expressed genes (DEG) analysis was performed using DESeq2 v1.24.0 with default sets with the Ballgown. Volcano plot was illustrated by using R v3.5.3, with the cutoff (more than four-fold changes with* p *< 0.05). The RNA-seq data was deposited in the Gene Expression Omnibus (GEO) database (accession number: GSE138069) and the comprehensive list of differentially expressed genes are listed in **Table S1**.

### Metabolite Extraction

*C. albicans* strains were inoculated to OD_600_ 0.2 in 50 mL of YPD medium for yeast condition or 50 mL of RPMI 1640 medium for hyphal condition. For yeast condition, the flask was incubated at 30°C for 3 h. For hyphal conditions, the cells were transferred into petri-dishes (20 mL per dish) and incubated in a static incubator at 37°C for 3 h. To harvest the yeast cells, the 50 mL of YPD was spun down at 13,000 x g for 10 min and was washed with cold PBS. The yeast cells were then transferred into chilled 1.5 mL tubes. To harvest the hyphal cells, the RPMI media was poured out and the adhered hyphal cells were washed with cold PBS. Using a cell scraper, the hyphal cells were dislodged from the bottom of the petri-dish and resuspended in cold PBS. The hyphal cells were then transferred into chilled 1.5 mL tubes. Harvested cells were resuspended in 700 μl of pre-chilled 40:40:20 (*vol:vol:vol*) buffer made of acetonitrile, methanol, and deionized water, respectively. The cells were mixed with 100 μl of 0.1 mm Zirconia beads (Biospec) and lysed in a bead beater programmed for 6800 rpm with eight 30 s cycles and 45 s pauses between cycles (Precellys Evolution) at 2°C. The cells were then centrifuged at 10,000 rpm for 10 min at 4°C and the supernatant was transferred to a Spin-X HPLC 0.2 μm nylon filter tube (COSTAR). The Spin-X tube was centrifuged at 10,000 rpm for 5 min at 4°C and the subsequent samples were stored in −80°C until ready for Liquid chromatography mass spectroscopy (LC-MS) (Lee et al., 2018; Lee et al., 2019).

### Liquid Chromatography Mass Spectrometry (LC-MS)

Extracted metabolites were separated on a Cogent Diamond Hydride Type C column (gradient 3) (Microsolve Technologies) and the mobile phase consisted of solution A (ddH2O with 0.2% formic acid) and solution B (acetonitrile with 0.2% formic acid). The mass spectrometer used was an Agilent Mass 6230 time of flight (TOF) coupled with an Agilent 1290 liquid chromatography (LC) system. Detected ions were deemed metabolites on the basis of unique accurate mass-retention time identifiers for masses exhibiting the expected distribution of accompanying isotopologs. The abundance of extracted metabolites was measured using Agilent Qualitative Analysis B. 08.00 software and Profinder B.0.800 software (Agilent Technologies) with a mass tolerance of <0.005 Da. All data obtained by metabolomics were the average of three independent samples of each condition tested. Analysis was performed on metabolite intracellular concentration per milligram of total proteins (IC/mg) taken from GC-MS. The normalized metabolomic data used in this study are listed in **Table S2**. The variance of metabolome data was analyzed by 2D Principal Component Analysis and heatmap using Clustvis (https://biit.cs.ut.ee/clustvis/) (Metsalu and Vilo, 2015). Statistical significance was calculated using GraphPad PRISM using a two-way ANOVA with HSD Tukey post-hoc analysis.

### Menadione Susceptibility Test

The susceptibility of various *C*. *albicans* strains to Menadione was tested by spot dilution assay. The cells were grown for overnight in YPD at 30°C and quantified. Serial 10-fold dilutions of the strains in 5 μl PBS (range 10^4^ to 10^1^ colony forming units (CFU) per spot) were spotted onto YPD agar containing 31.25 and 62.5 μM Menadione, as well as 2 M glycerol and 1 M NaCl. YPD agar with the same amount of DMSO in the 62.5 μM Menadione plate was used as a no drug control. The plates were incubated at 30°C for 48 h and photographed in UVP Gel Doc-It system (UVP, CA).

### 2′,7′-Dichlorofluorescin diacetate (DCFDA) Staining

Intracellular reactive oxygen species levels of various *C. albicans* strains were measured by 2′,7′-Dichlorofluorescin diacetate (DCFDA) staining (Du et al., 2015). *C. albicans* strains were suspended in RPMI 1640 medium and seeded in a flat-bottomed 96-well plate at 100,000 cells per well. The plates were incubated for 3 h at 37°C for hyphal induction. After incubation, the media was aspirated and the wells were resuspended in either 100 μl of sterile dH_2_O or 100 μl of 10 mM H_2_O_2_ and incubated for another 3 h at 37°C. Afterward, the cells were washed with PBS and resuspended in 100 μl of 20 μM DCFDA. The cells were incubated in the dark for 60 min, then washed and resuspended in 100 μl PBS. Cells positive for reactive oxygen species (ROS) were visualized using an EVOSmicroscope (Invitrogen) with a GFP filter.

## Results

### Overview of Transcriptome Acquisition

**Figure 1:**
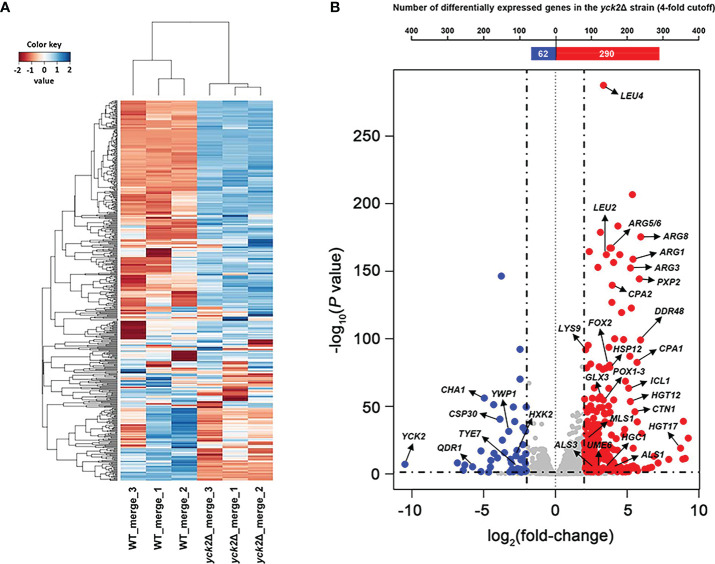
Transcriptomic profile of the *yck2*Δ strain. **(A)** Heat map visualization of the hierarchical clustering shows a distinct difference between the transcriptome of the wild type and the *yck2*Δ strains. **(B)** List of 352 differentially expressed genes in the *yck2*Δ strain were classified by log_2_ fold-change. Marked genes are related to carbon metabolism, amino acid metabolism, and hyphal formation.

The genes differentially expressed in the *yck2*Δ* *strain as compared to the wild type strain were identified by a comparative RNA-seq analysis. The raw sequencing data of the six samples were mapped to the *C. albicans *SC5314 genome (http://www.candidagenome.org). Quality control of the overall transcriptome acquisition was done by creating 2D principal component analysis (2D-PCA) plots (**Figure S1A**) and all2all plots (**Figure S1B**) using DEBrowser. In **Figure S1A**, respective wild type and *yck2*Δ replicates cluster well with each strain, indicating consistency between biological replicates. In **Figure S1B**, the correlation coefficient (rho) was calculated to measure the strength of association among biological replicates. The rho ranging 0.81 to 0.85 for the wild type strain and 0.89 to 0.93 for the *yck2*Δ strain suggested that the replicates of each strain showed similar transcriptome pattern. In contrast, the rho ranging 0.64 to 0.68 between the transcriptome of the wild type and the *yck2*Δ strains indicated that each strain exhibited a distinct transcriptome pattern. In **Figure 1A**, hierarchical clustering analysis revealed that the wild type and the *yck2*Δ* *strains showed markedly different transcriptome patterns between the two strains. Out of the 6263 annotated genes, a total of 885 genes were differentially regulated with statistical significance in the *yck2*Δ strain in comparison to the expression of genes in the wild type strain. Of these 885 genes, 673 genes were more than 2-fold upregulated, whereas 212 genes were more than 2-fold downregulated in the *yck2*Δ strain as compared to the wild type strain. Of these 885 genes, 359 genes had greater than a 4-fold change; 290 genes were more than 4-fold upregulated and 69 genes were more than 4-fold downregulated (**Figure 1B**).

### Genes Involved in Carbon Metabolism Are Differentially Expressed in the *yck2*Δ Strain

**Figure 2:**
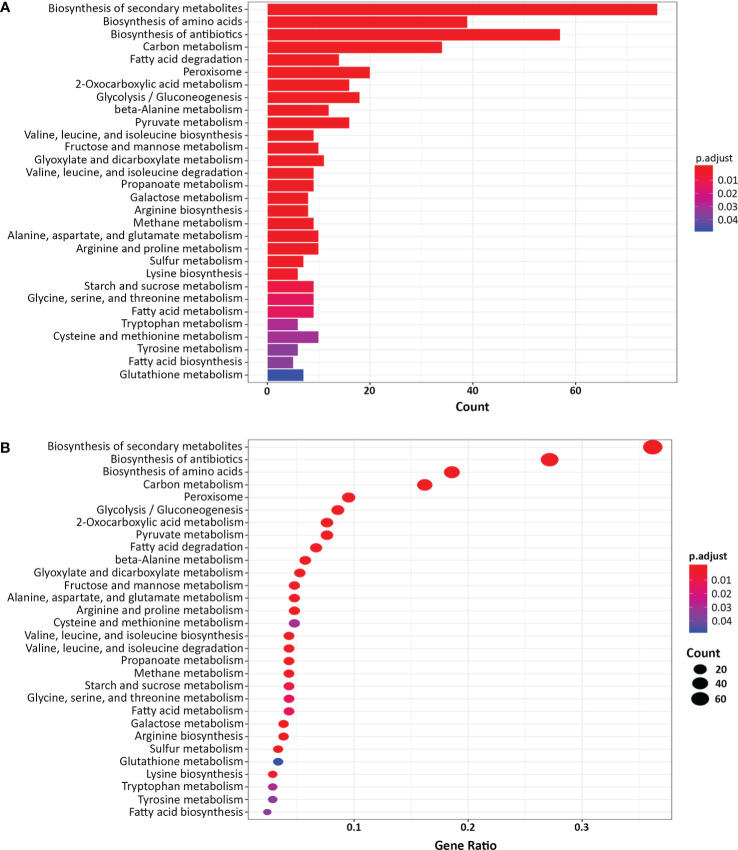
Gene Ontology (GO) term analysis of the differentially expressed genes in the *yck2*Δ strain. **(A)** List of 352 differentially expressed genes in the *yck2*Δ strain were functionally classified using DAVID (FC > 2 or FC <-2, padj < 0.05). Several overrepresented GO terms are related to amino acid and carbon metabolism. **(B)** Gene ratio of the GO categories.

Next, we classified the genes that were differentially expressed more than 4-fold in the *yck2*Δ strain compared to the wild type strain using Gene Ontology (GO) term analysis (DAVID, https://david.ncifcrf.gov). GO term analysis suggested that the genes involved in fatty acid beta-oxidation, glyoxylate cycle, peroxisome, and arginine biosynthetic process were upregulated in the *yck2*Δ strain when grown in yeast-inducing conditions, suggesting that *YCK2* is involved in carbon metabolism and amino acid biosynthesis (**Figure 2**). Among the genes involved in carbon metabolism, many of the upregulated genes are hexose transporters that are repressed by Mig1 in the presence of glucose (Fan et al., 2002; Sexton et al., 2007; Lagree et al., 2020) (**Table S1**). In contrast, the genes involved in glycolysis were downregulated, most notably *TYE7, PFK1*, and *PFK2*. Tye7 is a key transcription regulator of glycolysis that binds the promoters of glycolytic genes such as *PFK1* and *PFK2* that encode phosphofructokinase subunits (Askew et al., 2009). Phosphofructokinase irreversibly converts fructose-6-phosphate into fructose-1,6-bisphosphate, which is one of the critical regulatory steps of glycolysis (Berg JM and Stryer, 2002). In the RNA-seq data, downregulation of *PFK1/2* was consistent with downregulation of *TYE7*, clearly indicating that glycolysis is diminished in the *yck2*Δ strain. In addition, the genes involved in beta-oxidation (*PXP2*, *POX1-3, FOX2, POT1*) and the glyoxylate cycle (*ICL1, MLS1*) were upregulated in the *yck2*Δ strain. In *C. albicans*, beta-oxidation is important for metabolizing fatty acids into acetyl-CoA in the absence of preferred carbon sources such as glucose. Similarly, the glyoxylate cycle is a TCA cycle shunt that allows cells to assimilate simple carbon compounds, enabling *C. albicans* to grow and replenish TCA cycle metabolites when glucose is absent (Lorenz and Fink, 2001; Piekarska et al., 2006; Chew et al., 2019). Collectively, these results indicate that *YCK2* deletion elicits a glucose starvation response in *C. albicans* which matches with our hypothesis that CaYck2 is involved in glucose-utilizing signaling similar to ScYck1/2.

### The Hyphal Transition Program Is De-Repressed in the *yck2*Δ Strain

Many of the upregulated genes are hyphal specific genes (HSGs) (**Table 2** and **Table S1**). The RNA-seq data corroborated our previous results, showing upregulation of the transcription factor *UME6* along with the downstream adhesins *ALS1, ALS3*, and hyphal cell wall protein *HWP1* (Jung et al., 2017). Other upregulated HSGs included cell wall proteins (*RBT1, DDR48*) and secreted enzymes during tissue invasion (*SAP6, ECE1*). Upregulation of HSGs was also accompanied with downregulation of yeast-specific genes (*IFE2, TPO3, OPT9, YWP1, DAK2, YHB1*) with *YWP1* being a notable yeast-specific cell wall protein (Harcus et al., 2004). Similarly, genes involved in hyphal initiation (*NRG1*, *BRG1*) were differentially expressed. In the *yck2*Δ strain, expression of *NRG1* was downregulated while *BRG1* was upregulated. These results support the finding that Nrg1 transcriptionally represses HSGs and that BRG1 prevents Nrg1-mediated repression of HSGs (Lu et al., 2014). These results corroborate our prior findings and indicate derepression of the hyphal program in the *yck2*Δ strain.

**Table 2:** Differentially expressed genes in the *yck2*Δ strains as compared to the wild type strain.

Categorization	Gene Name	orf19_ID	Description (taken from literature and *candidagenome.org*)	Log2 Foldchange	P-value
*Glycolysis*	TYE7	orf19.4941	Transcriptional regulator of glycolytic genes; regulates PFK1 and PFK2	−3.02	3.14E-05
	PFK2	orf19.6540	Phosphofructokinase beta subunit	−2.35	1.02E-04
	PFK1	orf19.3967	Phosphofructokinase alpha subunit	−1.81	1.15E-02
	HXK2	orf19.542	Hexokinase 2; catalyzes first step of glycolysis	−3.13	6.62E-13
*Beta-Oxidation*	PXP2	orf19.1655	Putative acyl-CoA oxidase; putative peroxisome targeting signal	5.85	1.35E-147
	POX1-3	orf19.1652	Predicted acyl-CoA oxidase	2.45	2.53E-53
	FOX2	orf19.1288	3-hydroxyacyl-CoA epimerase	3.50	2.97E-81
	POT1	orf19.7520	Putative peroxisomal 3-oxoacyl CoA thiolase	2.57	7.05E-59
*Glyoxylate Cycle*	ICL1	orf19.6844	Isocitrate lyase; required for virulence in mice; induced upon macrophage engulfment	5.22	2.10E-56
	MLS1	orf19.4833	Malate synthase	2.76	2.58E-23
*Hyphal-Specific Genes (HSGs)*	BRG1	orf19.4056	Transcription factor; recruits Hda1p to promoters of hyphal-specific genes	1.62	4.57E-02
	UME6	orf19.1822	Activator of downstream hyphal genes	2.74	1.36E-04
	ALS3	orf19.1816	Cell wall adhesin; endothelial invasion	2.43	4.48E-02
	ALS1	orf19.5741	Cell-surface adhesin	3.10	1.67E-05
	HWP1	orf19.1321	Hyphal cell wall protein	1.28	3.76E-01
*Yeast-Specific Genes*	YWP1	orf19.3618	Secreted yeast wall protein	−3.86	3.54E-43
*Arginine Biosynthesis*	ARG1	orf19.7469	Argininosuccinate synthase	6.74	0.00E+00
	ARG3	orf19.5610	Putative ornithine carbamoyltransferase	5.23	9.60E-156
	ARG4	orf19.6689	Argininosuccinate lyase	3.90	9.02E-171
	ARG5/6	orf19.4788	Arginine biosynthetic enzyme	3.79	1.00E-170
*Arginine Degradation*	CAR1	orf19.3934	Arginase	−1.95	1.90E-41
	CAR2	orf19.5641	Ornithine aminotransferase	−2.46	2.48E-95
	PUT1	orf19.4274	Putative proline oxidase	3.55	9.62E-166
	PUT2	orf19.3974	Putative delta-1-pyrroline-5-carboxylate dehydrogenase	3.14	1.48E-182
*Nitric Oxide Detoxification*	YHB1	orf19.3707	Nitric oxide dioxygenase	−1.60	4.41E-02
*Oxidative Stress Response Genes*	CAT1	orf19.6229	catalase	2.44	6.90E-37
	SOD5	orf19.2060	superoxide dismutase	2.48	7.64E-03
	SOD4	orf19.2062	superoxide dismutase	2.50	5.96E-11

### *YCK2* Deletion Upregulates Oxidative Stress Response Genes

The transcriptome data also revealed that genes involved in the oxidative stress response were upregulated in the *yck2*Δ strain. *YCK2* deletion upregulated genes that encode catalase (*CAT1*), superoxide dismutase (*SOD4*, *SOD5*), and arginine biosynthetic genes (*ARG1, ARG3, ARG4, ARG5/6*) (**Table 2**). Upregulation of arginine biosynthetic genes has been shown to be a specificresponse of *C. albicans* upon exposure to ROS (Jimenez-Lopez et al., 2013). These results suggest two possibilities: (1) CaYck2 is involved in transcriptionally regulating oxidative stress response genes, or (2) deletion of *YCK2* leads to increased intracellular production of ROS. **Figure S2** shows that the *yck2*Δ strain is hypersusceptible to 62.5 μM menadione, a concentration which does not inhibit the wild type strain. Similarly, DCFDA staining shown in **Figure S3** indicates that the *yck2*Δ strain has a higher intracellular ROS level compared to the wild type strain. Together, these findings lean towards the latter possibility that a lack of CaYck2 alters intracellular ROS levels, leading to upregulation of oxidative stress response genes including ROS detoxifying enzymes and arginine biosynthetic genes.

### Overview of Metabolome in the Wild Type and *yck*2Δ Strains

Considering that many of the differentially expressed genes in the *yck2*Δ strain were related to metabolism, we followed up on our transcriptome analysis by measuring carbon metabolites with metabolomic analysis. Based on our finding that the *yck2*Δ strain is transcriptionally in a hyphal state, we hypothesized that the *yck2*Δ strain likely mimics a hyphal specific metabolome. Thus, we compared the metabolome of the wild type, *yck2*Δ, and *YCK2* complemented strains under yeast- and hyphal-inducing conditions. First, the overall metabolomic profiles of the three strains under each condition were compared by 2D-PCA and hierarchical clustering analysis.

**Figure 3:**
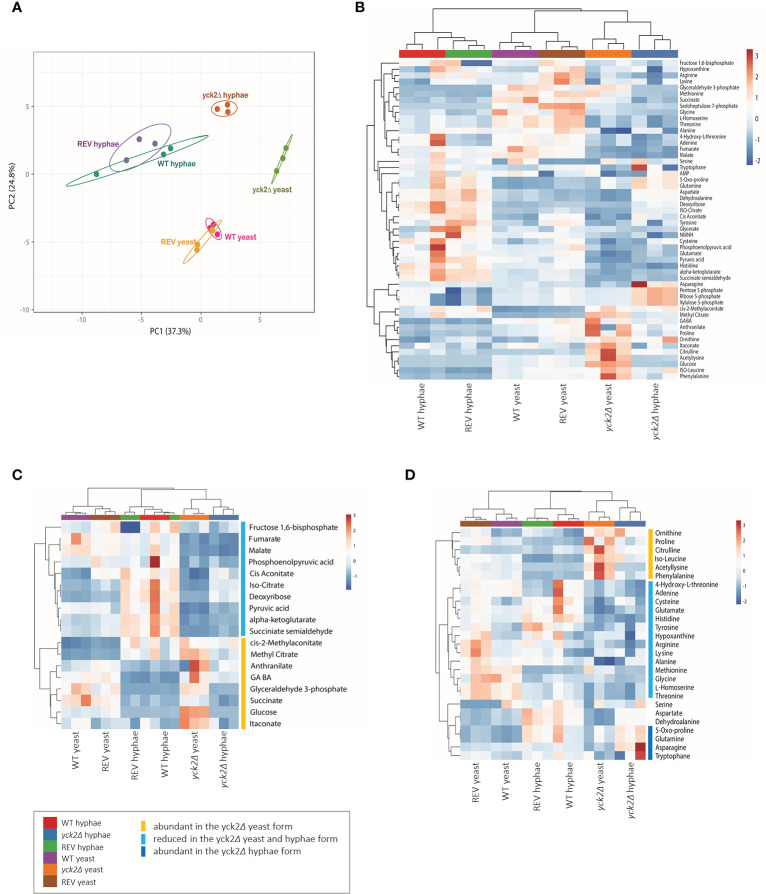
Metabolomic profile of the *C. albicans* yck2Δ strain. **(A)** 2D Principal-component analysis (PCA) of wild type, yck2Δ, and complemented strain metabolomes under yeast (YPD, 30°C) and hyphal (RPMI, 37°C) conditions. **(B)** Heatmap of metabolomes of various C. albicans cells grown under yeast and hyphal conditions. The colored bar on top of the heat maps indicates biological replicates of wild type (WT, purple and red), yck2Δ (orange and blue), and complemented (REV, brown and green) strains from yeast and hyphal conditions, respectively. **(C)** Hierarchical clustering of carbon metabolites. **(D)** Hierarchical clustering of amino acids. The colored bars on the right of the heatmaps indicate the metabolites with different abundance in the yck2Δ strain compared to the wild type strain.

2D-PCA analysis was performed to compare variations among the metabolites of the wild type, yck*2*Δ, and complemented strains grown under yeast and hyphal conditions (**Figure 3A**). The overall metabolome of the wild type (red for yeast, green for hyphae) and the *YCK2* complemented (yellow for yeast, purple for hyphae) strains clustered together, suggesting the metabolic activity of the complemented strain resembles that of the wild type strain. The clusters of yeast cells were located far from that of hyphal cells, suggesting that yeast and hyphal cells have distinct metabolomes. The overall pattern of the *yck2*Δ* *metabolomes (lime for yeast, brown for hyphae) did not cluster with either yeast or hyphal metabolomes of wild type strain, suggesting that the *yck2Δ* strain is somewhat variable and may include metabolites distinct from both wild type yeast and hyphal cells.

The heat map further revealed that the *yck2*Δ* *strain had significant metabolome differences compared to the wild type strain under both yeast and hyphal conditions (**Figure 3B** ). It is notable that the metabolome of the *yck2*Δ* *strain in yeast and hyphal conditions are clustered together, suggesting that the mutant strain had less variation between yeast and hyphal metabolomes. Supporting the transcriptome analysis data, many of the impacted metabolites are intermediates important for carbon metabolism (**Figure 3C**) and abundance of several amino acids (**Figure 3D** ) are also different between the wild type and the *yck2*Δ strains.

### Metabolome of Yeast and Hyphal Cells Have Differences in Carbon Metabolism and Alternative TCA Cycle Shunts

**Figure 4:**
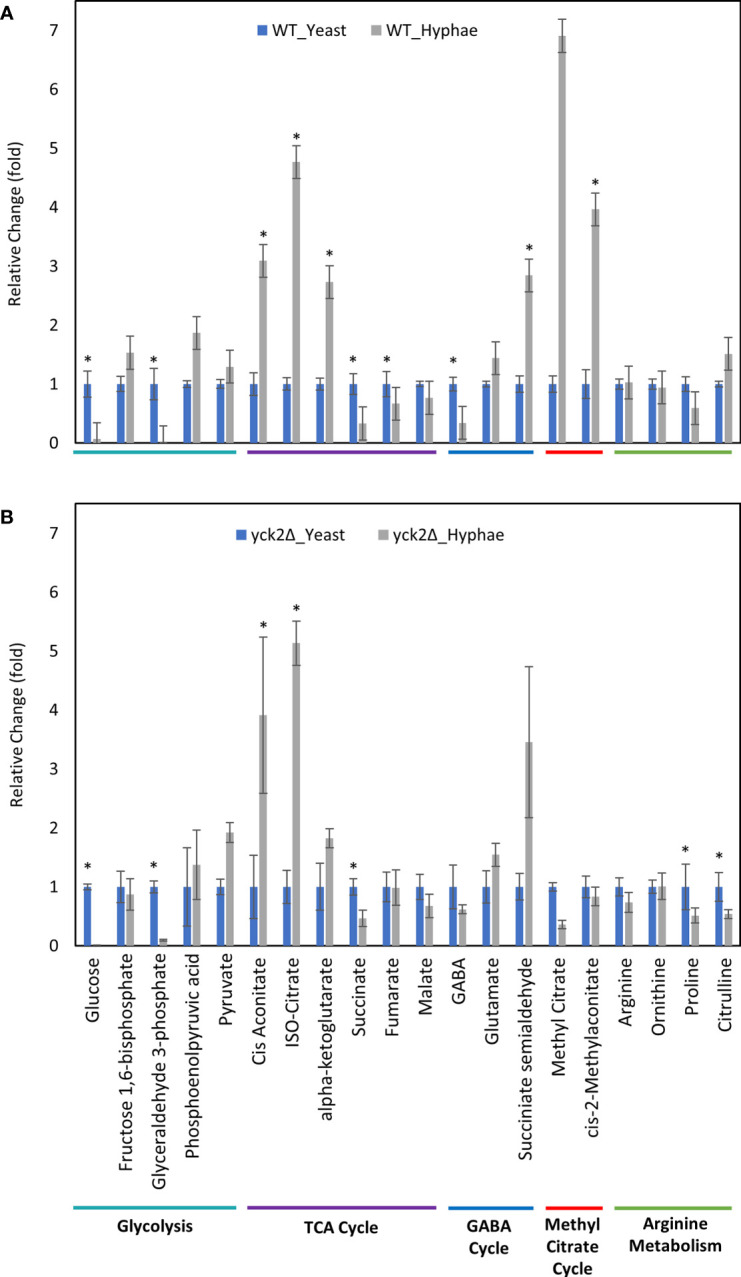
Carbon metabolites with differential abundance under yeast and hyphal conditions. **(A)** Relative abundance of wild type metabolites under yeast and hyphal conditions **(B)** Relative abundance of the *yck2Δ* metabolites under yeast and hyphal conditions. Intracellular concentration per milligram of total proteins (IC/mg) of each metabolite was normalized. Fold change was calculated relative to metabolite abundance in the yeast condition. (*p < 0.05) Statistical significance was tested by two-way ANOVA with HSD Tukey’s *post hoc* analysis.

Before closely looking at the metabolome of the *yck2Δ *strain, we first analyzed how the metabolome of the wild type strain differs in yeast and hyphae forms (**Figure 4A** ). In the wild type strain, hyphal cells had significantly reduced glycolytic metabolite abundances relative to yeast cells; glucose decreased by 15-fold and glyceraldehyde-3-phosphate decreased by 89-fold from their levels in yeast forms. Oxidative TCA cycle metabolite abundances were increased in the hyphal cells; cisaconitate increased by 3.09-fold, isocitrate by 4.77-fold, and α-ketoglutarate by 2.73-fold. Conversely, reductive TCA cycle metabolite abundances were reduced; succinate decreased by 3.03-fold and fumarate decreased by 1.49-fold from their yeast levels.

During yeast to hyphal transition, there is also a notable increase in methyl citrate, cis-2-methyl aconitate, and succinate semialdehyde, along with a decrease in gamma aminobutyric acid (GABA). From yeast to hyphae, methyl citrate increased by 6.91-fold, cis-2-methylaconitate increased by 3.96-fold, succinate semialdehyde increased by 2.84-fold, and GABA dropped by 2.94-fold (**Figure 4A**). In other organisms, these metabolites are part of the methyl citrate cycle and GABA degradation, both of which intersect with the TCA cycle as alternative shunts. The methyl citrate cycle has been documented in human pathogens such as the bacteria *Mycobacterium tuberculosis* and in the fungi *Paracoccidioides*, which rely on beta-oxidation when invading glucose-poor host niches (Eoh and Rhee, 2014; Santos et al., 2020). However, little is known about the methyl citrate cycle in *C. albicans*. The GABA degradation pathway is well conserved in eukaryotes and has been studied in other fungi such as *S. cerevisiae*, *Aspergillus nidulans*, and *Neurospora crassa*. Though GABA degradation is not well understood in *C. albicans, S. cerevisiae* orthologs of GABA degradation enzymes have been discovered in *C. albicans* (Bach et al., 2009; Cao et al., 2013).

### *YCK2* Deletion Causes Glucose Accumulation, a Depletion of TCA Cycle Intermediates and an Increase in Methyl Citrate Cycle and Arginine Degradation Intermediates

From the transcriptome data, our original prediction was that the *yck2*Δ strain would have a similar metabolome profile to wild type hyphal cells regardless of yeast or hyphal conditions. However, relative comparisons of the *yck2*Δ strain with the wild type revealed two patterns: (1) some hyphal-specific changes in the metabolome are still visible in the *yck2*Δ strain and that (2) the *yck2*Δ strain has changes in the metabolome that are distinct from either the yeast or hyphal wild type metabolomes.

Comparable to the wild type strain, the *yck2*Δ strain grown in hyphal conditions had reduced glycolytic metabolite abundances relative to cells grown under yeast conditions; glucose decreased by 174-fold and glyceraldehyde-3-phosphate decreased by 10-fold from their yeast grown counterparts (**Figure 4B**). Similarly, TCA cycle metabolites cisaconitate and isocitrate increased by 3.91-fold and 5.13-fold respectively, and succinate decreased by two-fold. These are patterns that were visible in the wild type yeast to hyphal switch, suggesting that these metabolic changes are independent of CaYck2 function.

**Figure 5:**
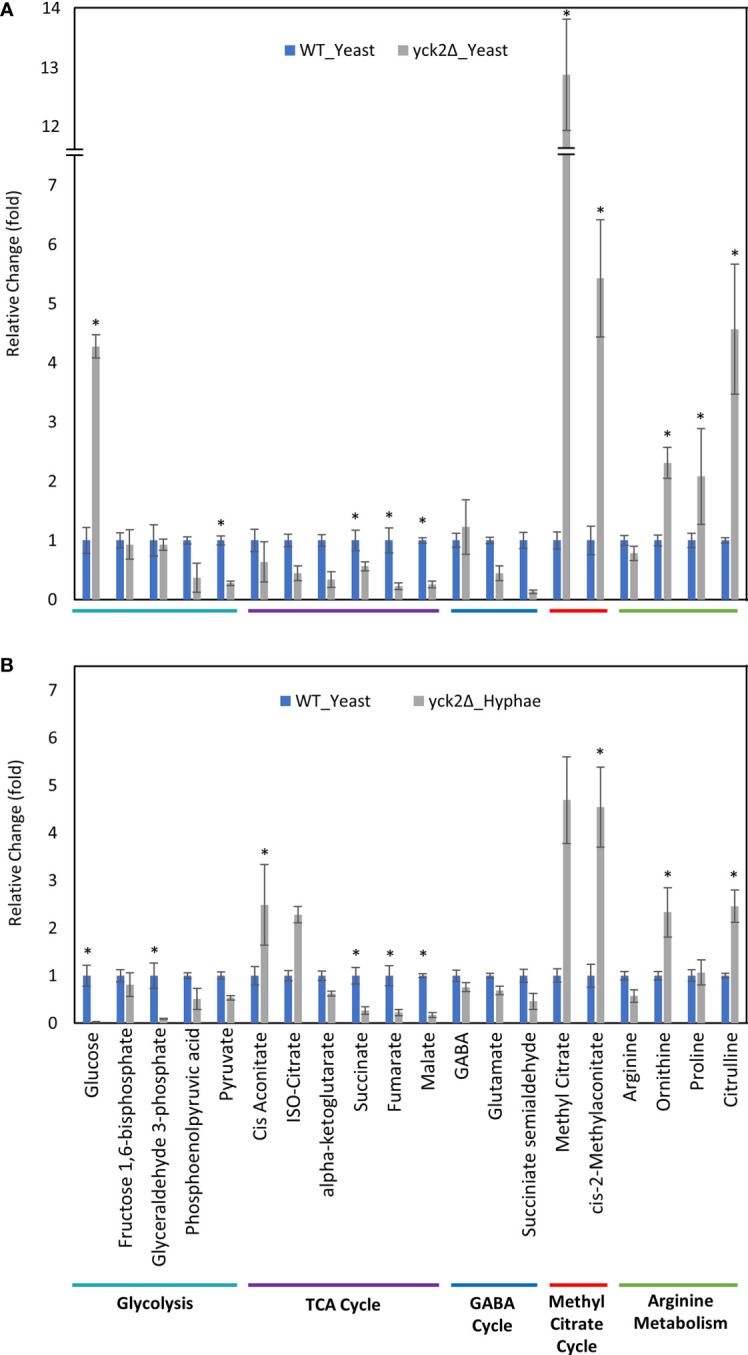
Carbon metabolites with differential abundance in the *yck2Δ* strain compared to the wild type yeast cells. **(A)** Relative abundance of *yck2Δ* yeast metabolites compared to wild type yeast metabolites. **(B)** Relative abundance of *yck2Δ* hyphae metabolites compared to wild type yeast metabolites. Intracellular concentration per milligram of total proteins (IC/mg) of each metabolite was normalized. Fold change was calculated relative to metabolite abundance in the wild type yeast cells. (*p < 0.05) Statistical significance was tested by two-way ANOVA with HSD Tukey’s *post hoc* analysis.

**Figure 6:**
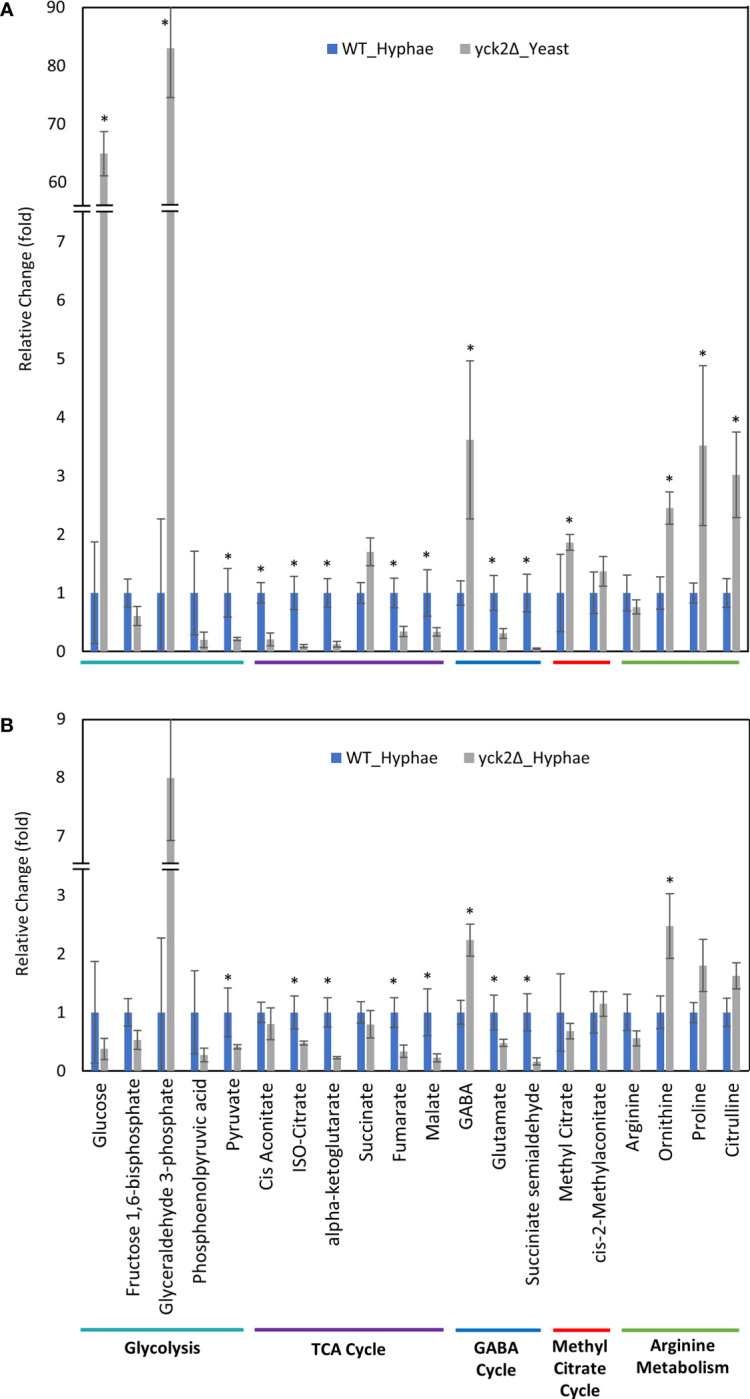
Carbon metabolites with differential abundance in the *yck2Δ* strain compared to the wild type hyphae cells. **(A)** Relative abundance of *yck2Δ* yeast metabolites compared to wild type hyphae metabolites. **(B)** Relative abundance of *yck2Δ* hyphae metabolites compared to wild type hyphae metabolites. Intracellular concentration per milligram of total proteins (IC/mg) of each metabolite was normalized. Fold change was calculated relative to metabolite abundance in wild type hyphae cells. (*p < 0.05) Statistical significance was tested by two-way ANOVA with HSD Tukey’s *post hoc* analysis.

In contrast, there are significant variations in the metabolome that are specific to the *yck2*Δ strain. The glucose abundance in *yck2*Δ strain grown in yeast condition was 4-fold and 65-fold higher than the wild type yeast and hyphal cells, respectively. This glucose accumulation coincided with a 3.60- and 4.67-fold decrease of pyruvate compared to the wild type yeast and hyphal cells, respectively (**Figures 5A** and **6A**). The accumulation of glucose in the *yck2*Δ strain in yeast condition is likely the result of upregulated hexose transporter genes and the downregulation of *PFK1/2* that would prevent the breakdown of glucose to pyruvate. However, it is unknown why this accumulation was not observed in the *yck2*Δ strain grown in hyphal conditions (**Figures 4B** and **5B**). While the *yck2*Δ strain under yeast conditions shows an 82-fold increase in Glyceraldehyde-3-phosphate when compared to wild type hyphal cells (**Figure 6A**), this large fold difference is due to the extremely low abundance of glyceraldehyde-3-phosphate in hyphal cells (**Figure S5**).

**Figure 7:**
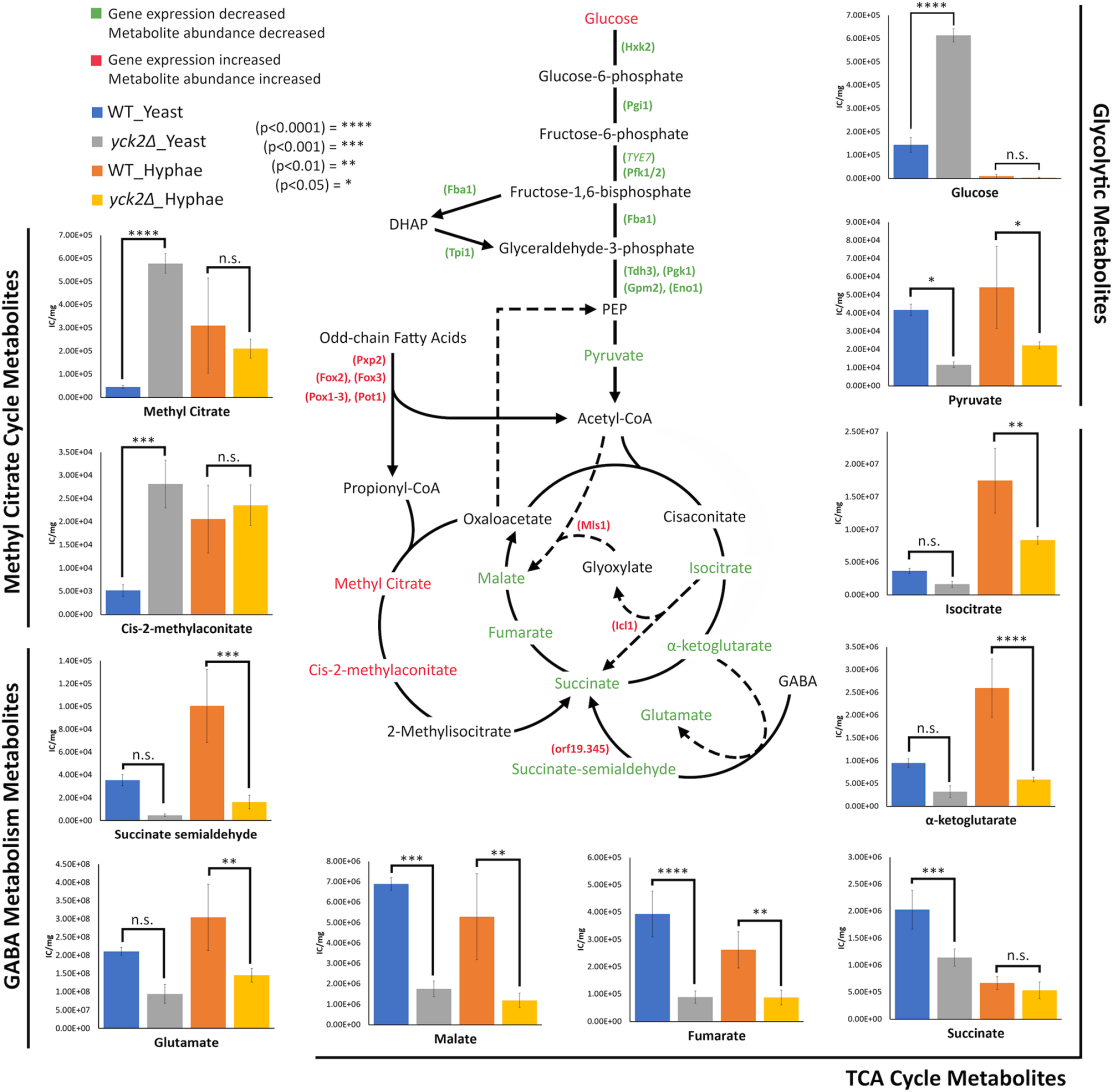
Altered carbon metabolites in the *yck2Δ* strain. Schematic of glycolysis, TCA cycle, the glyoxylate cycle, the methyl citrate cycle, and GABA degradation. Information on the methyl citrate cycle and GABA degradation were adapted from studies on other organisms (Bach et al., 2009; Cao et al., 2013; Eoh and Rhee, 2014). Metabolites and enzymes are color-coded based on results from transcriptome and metabolome analysis. Genes and the metabolites shown in green indicate the decreased transcription and metabolite abundance in the *yck2Δ* strain. Genes and the metabolites shown in red indicate the increased transcription and metabolite abundance in the *yck2Δ* strain. Metabolomics data is presented as intracellular concentration per milligram protein (IC/mg). Statistical significance was tested by two-way ANOVA with HSD Tukey’s *post hoc* analysis.

The *yck2*Δ strain also has a notable depletion of TCA cycle metabolites with abundances far lower than the wild type in either yeast or hyphal conditions (**Figures 5, 6**). For the *yck2*Δ strain in yeast conditions, succinate decreased by 1.78-fold, fumarate decreased by 4.40-fold, and malate decreased by 3.91-fold (**Figure 5A**). In hyphal conditions, the isocitrate decreased by 2.09-fold, α-ketoglutarate decreased by 4.40-fold, succinate decreased by 1.26-fold, fumarate decreased by 2.97-fold, and malate decreased by 4.44-fold (**Figure 6A** ). This depletion is most apparent with fumarate and malate where the *yck2*Δ strain has comparable abundances between yeast and hyphal conditions, but is significantly lower than in either yeast or hyphal wild type cells (**Figure 7**).

The high abundance of methyl citrate cycle metabolites may be associated with hyphal derepression in the *yck2*Δ strain. As shown in **Figure 4A**, methyl citrate and cis-2-methylaconitate was higher in hyphal conditions than yeast conditions in the wild type, suggesting that this increase is hyphae-specific. In contrast, **Figure 4B** shows that the *yck2*Δ strain has similar abundances of methyl citrate cycle metabolites between yeast and hyphal conditions. **Figure 5A** shows that the *yck2*Δ strain in either yeast or hyphal conditions consistently has a higher abundance of methyl citrate and cis-2-methylaconitate when compared to wild type yeast cells. Conversely, these differences are greatly diminished when the *yck2*Δ strain is compared to wild type hyphal cells (**Figures 6A, B** ). These differences are more visible when comparing the raw abundances of methyl citrate and cis-2-methylaconitate (**Figure 7**), suggesting that the increase in these metabolites is hyphae-specific in the wild type and that hyphal derepression in the *yck2*Δ strain is causing accumulation of these metabolites. In addition, the *yck2*Δ strain under yeast conditions also has a significant increase in arginine degradation intermediates ornithine, proline, and citrulline, suggesting that these changes are specific to the loss of *YCK2* (**Figures 5A**, **6A**).

## Discussion

*C. albicans* is equipped with complex regulatory mechanisms to detect environmental cues and is thus able to adapt its metabolism to a wide range of host niches. One critical metabolic adaptation is its ability to respond to carbon availability. *C. albicans* mutant strains with defective carbon metabolism are unable to colonize and infect host cells, or become hypersusceptible to phagocytic killing by macrophages (Barelle et al., 2006; Ries et al., 2018; Burgain et al., 2019). In addition, *C. albicans* strains with defective carbon metabolism also undergo perturbed morphogenic transition (Lorenz and Fink, 2001; Brown et al., 2006; Lagree et al., 2020), suggesting that the regulatory pathways governing the carbon metabolism might have direct link to morphogenesis of *C. albicans*. However, the regulatory networks between carbon metabolism and morphogenesis are not fully elucidated. In this study, we employed both transcriptomic and metabolomic analyses to describe the functions of CaYck2, which appears to link carbon metabolism and morphogenesis in *C. albicans*.

This study found that deletion of *YCK2* simulates a glucose starvation response at the transcriptional level with subsequent accumulation of glucose and depletion of TCA cycle metabolites. In *C. albicans*, the glucose repression pathway uses Mig1 and Mig2 as transcriptional repressors when glucose is abundant. Mig1 represses hexose transporters *HGT1, HGT2, HGT12, HGT13, HGT17, MAL31, *and *HXT5*. Mig2 represses expression of genes related to beta-oxidation such as *FOX2, POT1, HPD1, ALD6, ICL1, *and *MLS1* (Sexton et al., 2007; Otzen et al., 2014; Lagree et al., 2020). In contrast, the SRR pathway uses Rgt1 as a transcriptional repressor of HGTs when glucose is absent. Rgt1 represses hexose transporters *HGT1, HGT2*, *HGT7, HGT10, HGT12*, and *HXT5*, some of which are also controlled by Mig1 (Sexton et al., 2007; Otzen et al., 2014; Lagree et al., 2020). Rgt1 also represses glycolytic genes *TYE7* and *HXK2*. *HXK2* encodes hexokinase, which catalyzes the first step of glycolysis, which is another major point of regulation for glycolytic flux. Thus, Mig1 and Rgt1 work together to control the expression of HGTs to ensure that the proper hexose transporters are expressed in response to external glucose levels. Likewise, Mig2 and Rgt1 regulate carbon input by controlling glycolysis and alternative carbon metabolism pathways such as beta-oxidation. Many of the listed HGTs along with beta-oxidation genes are overexpressed in the *yck2Δ *strain, suggesting that Mig1 and Mig2 are inactive. Similarly, glycolytic genes *TYE7* and *HXK2* are repressed, suggesting that Rgt1 is active. In addition, Rgt1 does have binding sites on the *MIG1* promoter, which would explain the partial downregulation of *MIG1* in the transcriptome data (Sexton et al., 2007). These results suggested that CaYck2 deletion resulted de-repression of glucose-repressed genes within the Mig1/2-mediated glucose sensing pathway and downregulated glycolytic genes through the Rgt1-mediated SRR pathway (Robinson et al., 1993; Snowdon and Johnston, 2016). However, further studies are required to properly assess the role of CaYck2 in the SRR pathway.

Interestingly, the lack of CaYck2 induced a glucose starvation response and altered intracellular ROS levels closely mimicking what occurs during macrophage engulfment. Engulfment by macrophages causes an upregulation of beta-oxidation and the glyoxylate cycle, which allows *C. albicans* to sequester carbon from lipids and other simple molecules within the nutrient-deprived phagosome (Lorenz and Fink, 2001). The glyoxylate cycle in *C. albicans* is activated primarily in phagocytosed cells and are repressed by physiological levels of glucose. Upon escaping the phagosome *via* hyphae formation, *C. albicans *resumes glycolytic activities (Prigneau et al., 2003; Lorenz et al., 2004; Barelle et al., 2006; Chew et al., 2019). In addition, macrophages produce ROS to help kill pathogens captured withinthe phagosome. In *C. albicans*, exposure to ROS not only upregulates ROS detoxifying enzyme genes, but also upregulates arginine biosynthetic genes (Lorenz and Fink, 2001; Banerjee et al., 2008; Jimenez-Lopez et al., 2013), which then induces hyphae formation and allows *C. albicans* to escape from the macrophage phagosome. A good example is *ARG1* and how it is specifically upregulated in response to hydrogen peroxide. *ARG1* upregulation is lost when *C. albicans* is engulfed by macrophages that are unable to produce ROS (Jimenez-Lopez et al., 2013). Although it is unclear why the *yck2Δ *strain had increased intracellular ROS levels, it may explain the upregulation or ROS detoxifying enzyme genes and upregulation of arginine biosynthetic genes in the *yck2Δ* strain.

**Figure 8:**
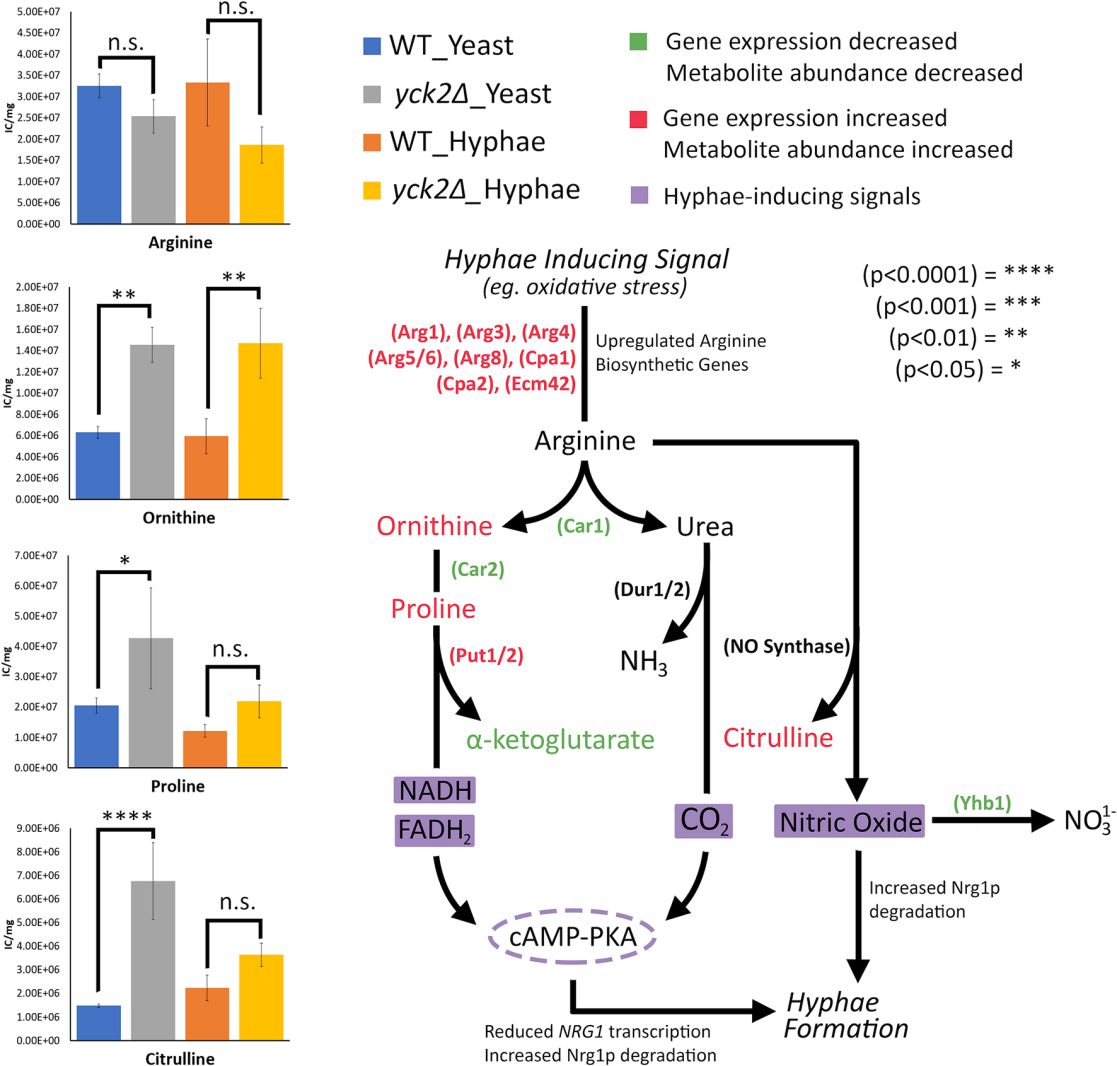
Arginine-dependent filamentation pathways and the abundance of the associated metabolites. Schematic for Dur1/2p, Put1/2p, and NO-dependent filamentation pathways. Metabolites and enzymes are color-coded based on results from transcriptome and metabolome analysis. Genes and the metabolites shown in green indicate decreased transcription and metabolite abundance in the yck2Δ strain. Genes and the metabolites shown in red indicate increased transcription and metabolite abundance in the yck2Δ strain. Highlighted in purple are the filamentation inducing signals. Metabolomics data is presented as intracellular concentration per milligram protein (IC/mg). Statistical significance was measured by two-way ANOVA with HSD Tukey’s post hoc analysis.

In addition, the importance of arginine catabolism in filamentation also provides insight to hyphal derepression in the *yck2Δ *strain. During macrophage engulfment, arginine is metabolized in either the Dur1/2-dependent or the Put1/2-dependent filamentation pathways to induce hyphae formation and macrophage escape (Jimenez-Lopez et al., 2013; Silao et al., 2019). These pathways rely on Car1 breaking down arginine into ornithine and urea. In Dur1/2-dependent filamentation, urea is broken down to generate CO_2_ as a morphogenic signal. In Put1/2-dependent filamentation, arginine is broken down to ornithine and proline. The Put1/2 pathway generates electron donors, NADH and FADH_2_, for the electron transport chain (ETC) which generate ATP as a morphogenic signal. Both the Dur1/2 and Put1/2 pathways induce filamentation through activation of the adenylate cyclase (cAMP-PKA) pathway (Jimenez-Lopez et al., 2013; Silao et al., 2019). Similarly, in nitric oxide (NO)-mediated filamentation, *C. albicans* cells maximize NO production by upregulating arginine biosynthetic genes and minimizing NO removal by downregulating *YHB1*, a gene encoding the primary nitric oxide detoxifier (Koch et al., 2018). Arginine can be broken down by nitric oxide synthase into NO and citrulline (**Figure 8**). Our results show changes in the expression of *CAR1*, *CAR2*, and *PUT1/2* as well as an accumulation of ornithine and proline in the *yck2*Δ* *strain, suggesting that the Put1/2 pathway may play a role in hyphal derepression. Similarly, our results show downregulation of *YHB1* and accumulation of citrulline, closely mirroring NO-mediated filamentation. Overall, the role of arginine catabolism in CaYck2-mediated filamentation must be further explored.

While the pathway by which CaYck2 regulates hyphae formation is still unknown, what is clear from the transcriptome data is that *YCK2* deletion impacts transcriptional regulators of the hyphal program. Results show that deletion of *YCK2* leads to a downregulation of the hyphal repressor *NRG1* and upregulation of the hyphae-specific transcription factor *UME*6, both of which are integral to hyphal initiation and elongation (Lu et al., 2011; Lu et al., 2014). Several studies have shown that *NRG1* overexpression locks *C. albicans* in the yeast state whereas its deletion leads to hyphal derepression and constitutive filamentation (Braun and Johnson, 1997; Braun et al., 2001; Murad et al., 2001). Ume6, a common downstream target for transcriptional regulators of hyphae formation such as Nrg1, controls the expression of HSGs and its deletion attenuates hyphal elongation (Banerjee et al., 2008; Banerjee et al., 2013). Thus, dysregulation of hyphal initiation and elongation would explain the constitutively filamentous phenotype of the *yck2*Δ* *strain.

It is important to address that, while the *yck2*Δ transcriptome resembles hyphae its metabolome is distinct from that of the wild type strain, which may explain why it forms pseudohyphae and not true hyphae. Studies have shown that pseudohyphae formation is controlled by a subset of HSGs and indicates a partial upregulation of the total hyphal program (Carlisle et al., 2009; Carlisle and Kadosh, 2013). It is thus possible that our transcriptome results are reflecting this partial upregulation of the hyphal program and would explain why the *yck2*Δ strain has a distinct metabolome from either yeast or hyphae of the wild type cells. We were unable to retrieve any literature that specifically studies the metabolome of pseudohyphal cells, making our findings the first of its kind. In addition, the *yck2*Δ* *strain itself has a distinct metabolome under yeast and hyphal conditions. This variation is most evident with glucose which accumulates in the *yck2*Δ* *strain under yeast conditions but not under hyphal conditions. This suggests that *yck2*Δ* *strain may have a distinct transcriptome between yeast and hyphal conditions that need to be further elucidated.

Interestingly, studies on *M. tuberculosis* draw surprising parallels with our transcriptome and metabolome results. *M. tuberculosis* relies on beta-oxidation to survive under glucose limiting conditions such as in the macrophage phagosome. As a result, propionyl-CoA, a toxic byproduct of odd-chain length fatty acid beta-oxidation, is generated, of which accumulation is toxic to *M. tuberculosis.* The methyl citrate cycle is a central pathway to metabolize propionyl-CoA first to methyl citrate, then to cis-2-methylaconitate, and then to methylisocitrate. Finally, methylisocitrate is converted to succinate by methylisocitrate lyase (David et al., 1993; Eoh and Rhee, 2014). Isocitrate lyases of the glyoxylate cycle (*ICL1* and *ICL2*) also function as methylisocitrate lyase in the methyl citrate cycle. A constitutively active methyl citrate cycle in *ICL*-deficient *M. tuberculosis* results in the dead-end accumulation of methyl citrate and cis-2-methylaconitate with subsequent depletion of TCA cycle intermediates (Eoh and Rhee, 2014). These results mirror closely what we see in the *yck2*Δ* *strain. Our results found that the *yck2*Δ* *strain accumulated methyl citrate and cis-2-methylaconitate, which suggests the existence of the methyl citrate cycle in *C. albicans*. However, this is in stark contrast to a previous finding that *C. albicans* does not have orthologues for methyl citrate cycle enzymes and does not show methylcitrate synthase activity (Otzen et al., 2014). Considering that the methyl citrate cycle in *C. albicans* is understudied, our results warrant further investigation.

Lastly, there are a few prior studies on the metabolome of *C. albicans* hyphae to compare with our wild type metabolome. Han *et al.* reported the metabolome profiles of *C. albicans* hyphal cells using three different hyphae inducing media: Lee’s medium, MPA, medium, and serum (Han et al., 2012). Their results suggest that certain changes in the metabolome are dependent on hyphae formation and not media. However, the metabolic changes they observed do not align with our findings, likely due to our use of RPMI 1640 medium for hyphal induction. This indicates that metabolic changes from yeast to hyphae have much more media-dependent variation than is currently known and highlights a need for further metabolic studies. This also indicates that the metabolome changes highlighted in **Figure 4** includes changes that are media-specific, thus requiring further studies to parse what changes are specific to the yeast to hyphal transition.

In summary, our transcriptome results show a carbon starvation profile, upregulation of oxidative stress genes including arginine biosynthetic genes, and de-repression of the hyphal program. Metabolomics shows depletion of TCA cycle metabolites and an increase in arginine degradation metabolites. Together, these findings suggest that CaYck2 regulates carbon metabolism and the oxidative stress response. This is corroborated by other studies that have highlighted the role of CaYck2 in metabolic adaptability in response to varying conditions such as hypoxia (Burgain et al., 2019). Because of how closely our results mimic the *C. albicans* response to macrophage engulfment, the importance of arginine catabolism in CaYck2-mediated filamentation needs to be further explored. In addition, results show accumulation of methyl citrate cycle metabolites which also parallels what is observed in *M. tuberculosis* when the bacterium survives intracellularly within macrophages. Further studies are needed to explore the existence of the methyl citrate cycle in *C. albicans* as our results are in direct contrast with previous findings (Otzen et al., 2014). Taken together, we discovered that Yck2 is an integral piece of carbon metabolism and morphogenesis of *C. albicans. C. albicans* is still a significant burden on human health and elucidating its complex virulence mechanisms must remain an ongoing endeavor, which is important for developing new therapeutics to combat infection.

## Data Availability Statement

The RNA-seq data is available in the Gene Expression Omnibus database(accession number: GSE138069) and the metabolome data is included in the **Supplementary Table 2**.

## Author Contributions

HP, HE, and Y-SB conceived and designed the experiments. KL, Y-SS, and S-RY conducted the RNA transcriptome profiling experiments and analyzed the transcriptome data. KL and JL conducted the metabolome profiling experiments and analyzed the metabolome data. KL and HP drafted the manuscript and KL, HP, HE, and Y-SB critical revised the manuscript for important intellectual content. All authors contributed to the article and approved the submitted version.

## Funding

Research reported in this publication was supported by the National Institute of General Medical Sciences of the National Institute of Health under Award Number R25GM061331, and the College of Natural and Social Sciences at California State University Los Angeles [NSS Research and Scholarship Award 2018]. This work was partly supported by the Strategic Initiative for Microbiomes in Agriculture and Food funded by the Ministry of Agriculture, Food and Rural Affairs (grant 916006-2 and 918012-4 to Y-SB) and, in part, by National Research Foundation of Korea grants (grants 2016R1E1A1A01943365 and 2018R1A5A1025077 to Y-SB) from the Ministry of Science and ICT.

## Conflict of Interest

The authors declare that the research was conducted in the absence of any commercial or financial relationships that could be construed as a potential conflict of interest.

## Supplementary Material

Supplementary Table 1 Comprehensive list of differentially expressed genes in the yck2Δ strain relative to the wild-type strain.

Supplementary Table 2 List of normalized metabolome data used in this study.

Supplementary Figure 1 Transcriptome data evaluation **(A)** Principal component analysis plots. Red dots and green dots indicate three biological replicates of the wild type and the yck2Δ strain, respectively. **(B)** All2all plots with correlation coefficient (rho) measuring the strength of correlation between transcriptomes. There is more similarity between biological replicates (GREEN) than comparisons between the wild type and the yck2Δ strain (BLUE).

Supplementary Figure 2 Menadione susceptibility test. Serial 10-fold dilutions of the indicated strains (10^4^ to 10^1^ cells per spot from left to right one each picture) grown on YPD with 31.25 μM and 62.5 μM Menadione. YPD with DMSO was used as a negative control for the menadione susceptibility test. YPD with 2 M glycerol and 1 M NaCl were included to demonstrate that the *yck2Δ* strain is resistant to other stresses.

Supplementary Figure 3 Detection of the intracellular reactive oxygen species by DCFDA staining. Strains were grown in hyphal conditions (RPMI, 37°C) with and without hydrogen peroxide and stained with DCFDA. Images were captured at 20x and the side bar indicates 20 μm. Without the addition of hydrogen peroxide, the *yck2Δ* strain has a visible fluorescent signal compared to the wild type strain.

Supplementary Figure 4 Fold-change comparison of the wild type and the complemented strain metabolomes in yeast and hyphal conditions. **(A)** Comparison of the yeast metabolome of the wild type and the complemented strains. **(B)** Comparison of the hyphal metabolome of the wild type and the complemented strains. Intracellular concentration per milligram protein (IC/mg) of each metabolite was converted to fold change relative to the abundance of metabolites in the wild type strain. (* p<0.05) Statistical significance was measured by two-way ANOVA with HSD Tukey’s *post hoc* analysis.

Supplementary Figure 5 Raw IC/mg results for glycolysis metabolites. Displays the intracellular concentration per milligram protein of glycolysis intermediates that were not shown in **Figure 7**. Statistical significance was measured by two-way ANOVA with HSD Tukey’s *post hoc* analysis.
