# Supplementary material for: Transcriptomic and Metabolomic Analysis Revealed Roles of Yck2 in Carbon Metabolism and Morphogenesis of Candida albicans
Source: Front Cell Infect Microbiol. 2021 Mar 16;11:636834. doi: 10.3389/fcimb.2021.636834 (PMC8008151; doi:10.3389/fcimb.2021.636834)
Supplement: Supplementary Table 1 — Comprehensive list of differentially expressed genes in the yck2Δ strain relative to the wild-type strain. [file Table_1.pdf]

Supplemental Table S1 - Comprehensive list of differentially expressed genes in the *yck2* Δ strain relative to the wild-type strain

Categorization	Gene Name	orf19_ID	Description (taken from literature and *candidagenome.org*)	Log2 Foldchange	P-value
Glucose Repressed Genes and Hexose Transporters	HGT1	orf19.4527	High-affinity MFS glucose transporter	2.24	1.11E-09
	HGT2	orf19.3668	Putative MFS glucose transporter	4.75	1.36E-102
	HGT4	orf19.5962	High-affinity glucose sensor; part of the SRR pathway; overexpression causes hyperfilamentation	1.62	1.61E-10
	HGT9	orf19.644	Putative glucose transporter	6.50	6.62E-06
	HGT12	orf19.7094	Expressed at low (0.2%) and high (5%) glucose concentrations	5.54	9.82E-49
	HGT13	orf19.7093	Predicted sugar transporter, involved in glycerol utilization	2.89	4.52E-28
	HGT17	orf19.4682	Putative glucose transporter; induced at low glucose concentrations	8.72	2.13E-21
	HGT19	orf19.5447	Putative MFS glucose/myo-inositol transporter; Responds to Hog1 stress response	3.01	1.25E-36
	HXT5	orf19.4384	Putative hexose transporter; colony morphology-related gene regulation; possibly essential	2.99	6.51E-28
	MAL31	orf19.3981	Putative high-affinity maltose transporter	3.02	1.78E-04
Glycolysis	TYE7	orf19.4941	Transcriptional regulator of glycolytic genes; regulates PFK1 and PFK2	-3.02	3.14E-05
	PFK2	orf19.6540	Phosphofructokinase beta subunit	-2.35	1.02E-04
	PFK1	orf19.3967	Phosphofructokinase alpha subunit	-1.81	1.15E-02
	HXK2	orf19.542	Hexokinase 2; catalyzes first step of glycolysis	-3.13	6.62E-13
	PGK1	orf19.3651	Phosphoglycerate kinase	-1.49	3.72E-05
	GPM2	orf19.1067	Putative phosphoglycerate mutase	-1.67	3.11E-04
	ENO1	orf19.395	Enolase	-1.30	2.66E-05
	TDH3	orf19.6814	Glyceraldehyde-3-phosphate dehydrogenase	-1.39	3.44E-06
	TPI1	orf19.6745	Triose-phosphate isomerase	-1.10	1.16E-03
	FBA1	orf19.4618	Fructose-bisphosphate aldolase	-1.68	1.48E-06
Beta-Oxidation	PXP2	orf19.1655	Putative acyl-CoA oxidase; putative peroxisome targeting signal	5.85	1.35E-147
	POX1-3	orf19.1652	Predicted acyl-CoA oxidase	2.45	2.53E-53
	FOX3	orf19.1704	Putative peroxisomal 3-oxoacyl CoaA thiolase	2.45	2.16E-84
	FOX2	orf19.1288	3-hydroxyacyl-CoA epimerase	3.50	2.97E-81
	POT1	orf19.7520	Putative peroxisomal 3-oxoacyl CoA thiolase; transcript regulated by Nrg1p and Mig1p	2.57	7.05E-59
	HPD1	orf19.5565	3-hydroxypropionate dehydrogenase; involved in degradation of toxic propionyl-CoA	5.10	2.35E-66
	ALD6	orf19.742	Putative aldehyde dehydrogenase	5.41	2.49E-162
Glyoxylate Cycle	ICL1	orf19.6844	Isocitrate lyase; glyoxylate cycle enzyme; required for virulence in mice; induced upon phagocytosis by macrophage; Pex5-dependent peroxisomal localization	5.22	2.10E-56
	MLS1	orf19.4833	Malate synthase; glyoxylate cycle enzyme; strong oxidative stress induced	2.76	2.58E-23
Acetyl Transport	CTN1	orf19.4551	carnitine O-acetyltransferase	5.71	3.96E-41
	CTN3	orf19.2809	carnitine O-acetyltransferase	3.48	9.14E-24

GABA Metabolism	CAALFM_C303470WA	orf19.345	Succinate semialdehyde dehydrogenase; for utilization of gamma-aminobutyrate (GABA) as a nitrogen source; part of GABA and glutamate degradation pathways	2.20	7.98E-26
Hyphal-Specific Genes (HSGs)	BRG1	orf19.4056	Transcription factor; recruits Hda1p to promoters of hyphal-specific genes	1.62	4.57E-02
	UME6	orf19.1822	Activator of downstream hyphal genes; expression is dependent on Brg1 mediated recruitment of Hda1 to UME6 promoter	2.74	1.36E-04
	ALS3	orf19.1816	Cell wall adhesin; epithelial adhesion, endothelial invasion	2.43	4.48E-02
	ALS1	orf19.5741	Cell-surface adhesin; adhesion, virulence	3.10	1.67E-05
	HWP1	orf19.1321	Hyphal cell wall protein	1.28	3.76E-01
	ECE1	orf19.3374	GPI-linked cell wall protein	2.18	1.54E-01
	HGC1	orf19.6028	Hypha-specific G1 cyclin-related protein involved in regulation of morphogenesis, biofilm formation; required for virulence in mice; regulated by Nrg1, Tup1	2.67	2.54E-03
	RBT1	orf19.1327	Cell wall protein with similarity to Hwp1; required for virulence; serum, hyphal and alkaline induced	3.77	1.15E-06
	RBT4	orf19.6202	TUP1 repressed; Pry family protein; required for virulence in mouse systemic/rabbit corneal infections	2.14	6.75E-32
	ECM331	orf19.4255	GPI-linked cell wall protein	3.27	5.28E-52
	SAP6	orf19.5542	Biofilm-specific aspartyl protease; expressed during hyphal growth, oral carriage, infection; virulence role affecte by URA3	2.18	6.59E-06
	DDR48	orf19.4082	Immunogenic stress-associated protein; Hog1 repressed	5.93	2.78E-102
	IHD2	orf19.6021	Protein of unknown function; induced in low iron; Spider biofilm induced	2.66	2.89E-14
	ARO10	orf19.1847	Aromatic decarboxylase; Ehrlich fusel oil pathway of aromatic alcohol biosynthesis	-2.93	2.63E-52
	RNH1	orf19.5563	Ribonuclease H (RNAse H)	2.15	3.00E-51
	GLG2	orf19.7434	Putative self-glucosylating initiator of glycogen synthesis	3.58	5.86E-20
	CSP37	orf19.2531	Hyphal cell wall protein; expressed in yeast and hyphae; hyphal downregulated	2.57	8.76E-23
Yeast-Specific Genes (YSGs)	IFE2	orf19.5288	Putative alcohol dehydrogenase; yeast-enriched transcript; Efg1-regulated; induced by Hog1	-3.17	1.09E-02
	TPO3	orf19.4737	Putative polyamine transporter; decreased expression in hyphae vs yeast-form cells; regulated by Nrg1	-2.38	1.75E-02
	OPT9	orf19.2584	Probable pseudogene similar to fragments of OPT1 oligopeptide transporter gene; transcriptionally induced upon phagocytosis by macrophage	-2.10	1.42E-09
	YWP1	orf19.3618	Secreted yeast wall protein	-3.86	3.54E-43
	DAK2	orf19.4777	Putative dihydroxyacetone kinase; repressed by yeast-hypha switch	-2.55	2.04E-03
	MEP1	orf19.1614	Ammonium permease; hyphal downregulated	2.23	6.95E-15
	NRG1	orf19.7150	Transcription factor; represses hyphal-specific genes	-1.38	8.31E-05
	FM_C5021	orf19.4216	Putative heat shock protein; decreased expression in hyphae	3.78	1.70E-83

Arginine Biosynthesis	ARG1	orf19.7469	Argininosuccinate synthase; arginine synthesis	6.74	0.00E+00
	ARG3	orf19.5610	Putative ornithine carbamoyltransferase; Gcn4-regulated	5.23	9.60E-156
	ARG4	orf19.6689	Argininosuccinate lyase, catalyzes the final step in the arginine biosynthesis pathway	3.90	9.02E-171
	ARG5/6	orf19.4788	Arginine biosynthetic enzyme; processed in S. cerevisiae into 2 polypeptides with acetylglutamate kinase (Arg6) activity and acetylglutamate-phosphate reductase (Arg5) activity	3.79	1.00E-170
	ARG8	orf19.3770	Putative acetylornithine aminotransferase	5.95	4.06E-179
	CPA1	orf19.4630	Putative carbamoyl-phosphate synthase subunit	5.19	3.55E-90
	CPA2	orf19.3221	Putative arginine-specific carbamoylphosphate synthetase	3.96	6.58E-143
	ECM42	orf19.6500	Ornithine acetyltransferase	3.31	2.36E-80
	GDH3	orf19.4716	NADP-glutamate dehydrogenase	3.40	9.09E-48
Arginine Degradation	CAR1	orf19.3934	Arginase	-1.95	1.90E-41
	CAR2	orf19.5641	Ornithine aminotransferase	-2.46	2.48E-95
	PUT1	orf19.4274	Putative proline oxidase	3.55	9.62E-166
	PUT2	orf19.3974	Putative delta-1-pyrroline-5-carboxylate dehydrogenase	3.14	1.48E-182
Nitric Oxide Detoxification	YHB1	orf19.3707	Nitric oxide dioxygenase	-1.60	4.41E-02
Oxidative Stress Response Genes	CAT1	orf19.6229	catalase	2.44	6.90E-37
	SOD5	orf19.2060	superoxide dismutase; induced by hyphal growth, osmotic/oxidative stress	2.48	7.64E-03
	SOD4	orf19.2062	superoxide dismutase	2.50	5.96E-11
