# Supplementary figures and images for: Transcriptomic and Metabolomic Analysis Revealed Roles of Yck2 in Carbon Metabolism and Morphogenesis of Candida albicans
Source: Front Cell Infect Microbiol. 2021 Mar 16;11:636834. doi: 10.3389/fcimb.2021.636834 (PMC8008151; doi:10.3389/fcimb.2021.636834)

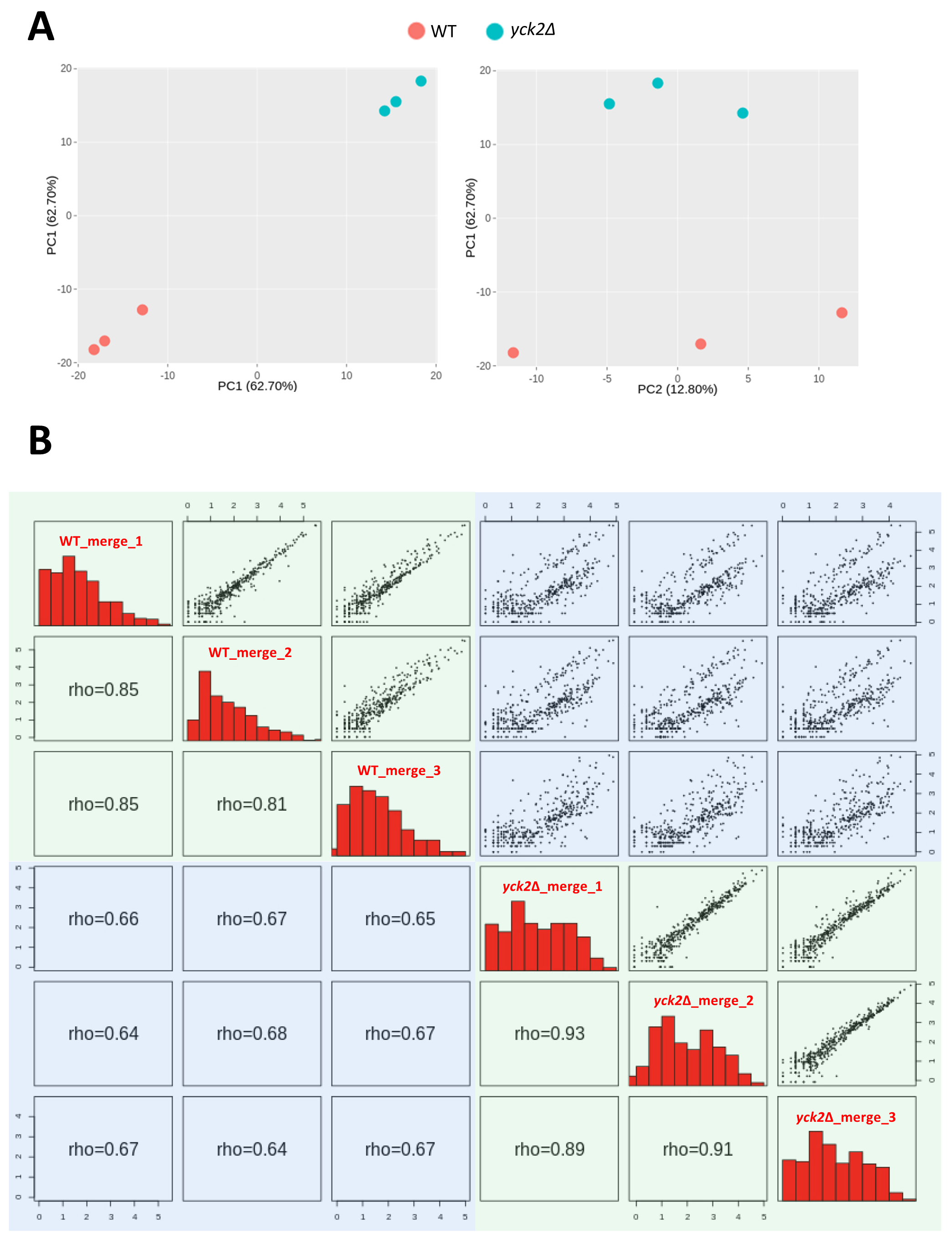

Supplement: Supplementary Figure 1 — Transcriptome data evaluation (A) Principal component analysis plots. Red dots and green dots indicate three biological replicates of the wild type and the yck2Δ strain, respectively. (B) All2all plots with correlation coefficient (rho) measuring the strength of correlation between transcriptomes. There is more similarity between biological replicates (GREEN) than comparisons between the wild type and the yck2Δ strain (BLUE). [file Image_1.tif]

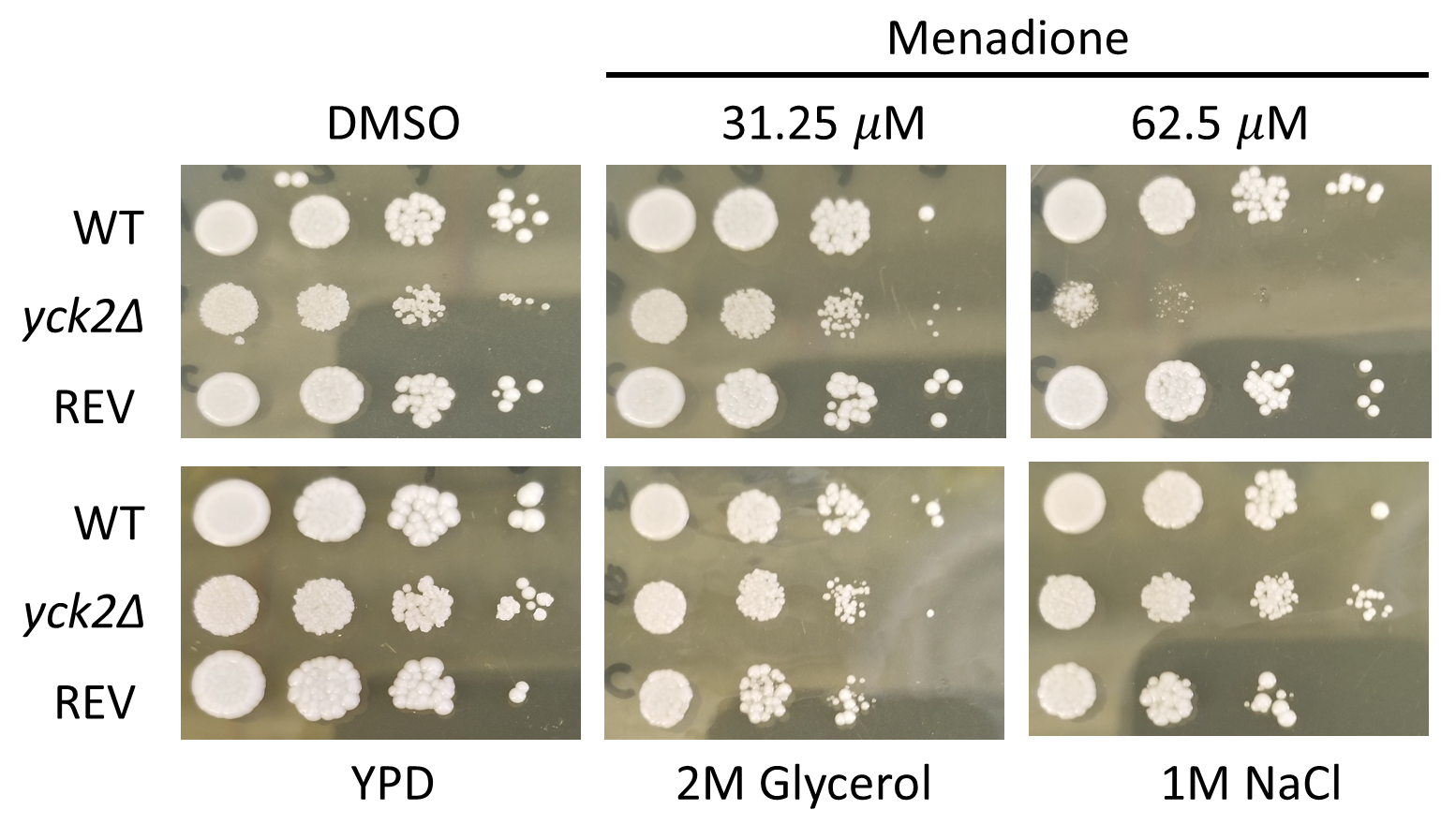

Supplement: Supplementary Figure 2 — Menadione susceptibility test. Serial 10-fold dilutions of the indicated strains (104 to 101 cells per spot from left to right one each picture) grown on YPD with 31.25 μM and 62.5 μM Menadione. YPD with DMSO was used as a negative control for the menadione susceptibility test. YPD with 2 M glycerol and 1 M NaCl were included to demonstrate that the yck2Δ strain is resistant to other stresses. [file Image_2.tif]

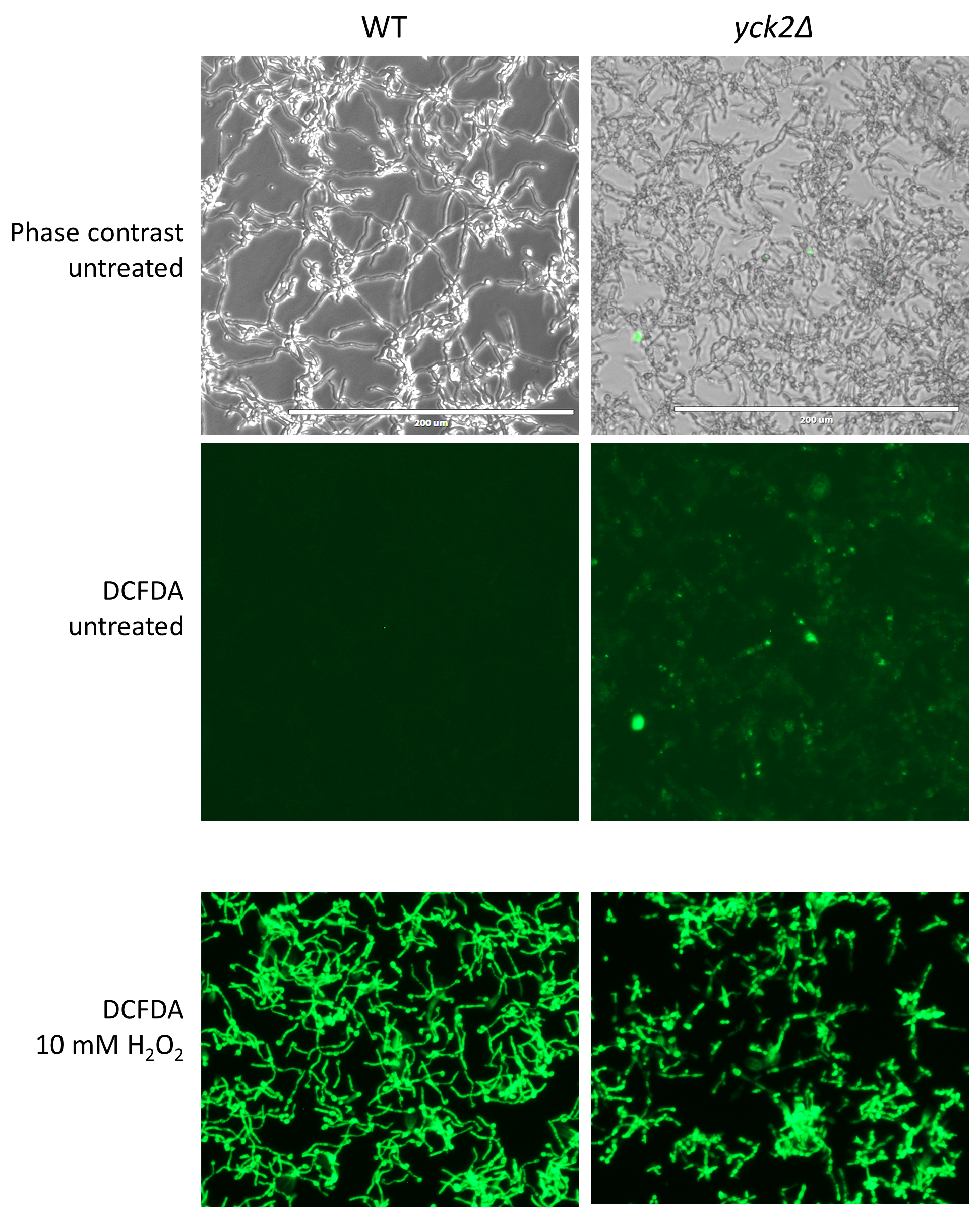

Supplement: Supplementary Figure 3 — Detection of the intracellular reactive oxygen species by DCFDA staining. Strains were grown in hyphal conditions (RPMI, 37°C) with and without hydrogen peroxide and stained with DCFDA. Images were captured at 20x and the side bar indicates 20 μm. Without the addition of hydrogen peroxide, the yck2Δ strain has a visible fluorescent signal compared to the wild type strain. [file Image_3.tif]

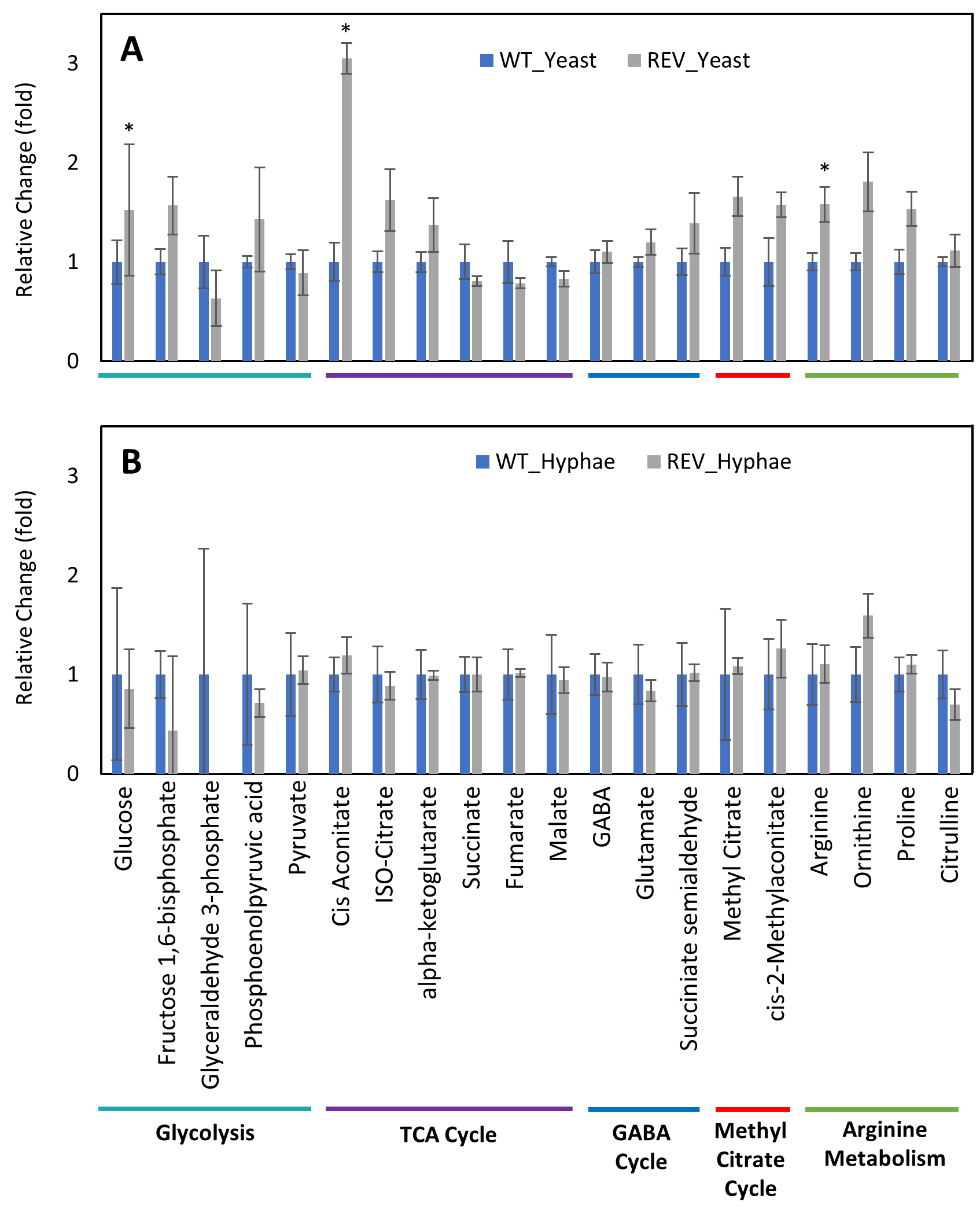

Supplement: Supplementary Figure 4 — Fold-change comparison of the wild type and the complemented strain metabolomes in yeast and hyphal conditions. (A) Comparison of the yeast metabolome of the wild type and the complemented strains. (B) Comparison of the hyphal metabolome of the wild type and the complemented strains. Intracellular concentration per milligram protein (IC/mg) of each metabolite was converted to fold change relative to the abundance of metabolites in the wild type strain. (* p<0.05) Statistical significance was measured by two-way ANOVA with HSD Tukey’s post hoc analysis. [file Image_4.tif]

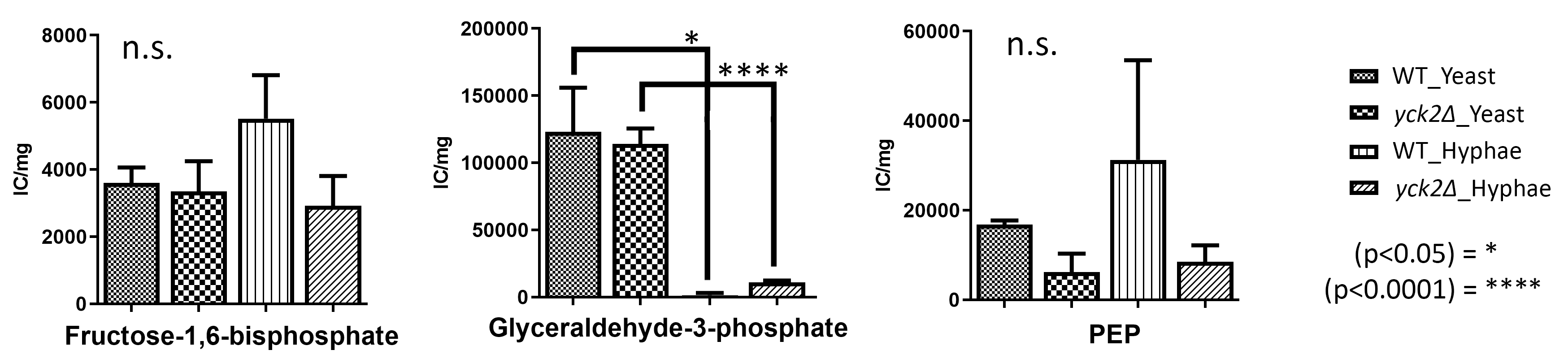

Supplement: Supplementary Figure 5 — Raw IC/mg results for glycolysis metabolites. Displays the intracellular concentration per milligram protein of glycolysis intermediates that were not shown in Figure 7 . Statistical significance was measured by two-way ANOVA with HSD Tukey’s post hoc analysis. [file Image_5.tif]
